# MVA-based vaccine candidates encoding the native or prefusion-stabilized SARS-CoV-2 spike reveal differential immunogenicity in humans

**DOI:** 10.1038/s41541-023-00801-z

**Published:** 2024-01-26

**Authors:** Leonie Mayer, Leonie M. Weskamm, Anahita Fathi, Maya Kono, Jasmin Heidepriem, Verena Krähling, Sibylle C. Mellinghoff, My Linh Ly, Monika Friedrich, Svenja Hardtke, Saskia Borregaard, Thomas Hesterkamp, Felix F. Loeffler, Asisa Volz, Gerd Sutter, Stephan Becker, Christine Dahlke, Marylyn M. Addo

**Affiliations:** 1 Institute for Infection Research and Vaccine Development (IIRVD), University Medical Centre Hamburg-Eppendorf, Hamburg, Germany; 2 Department for Clinical Immunology of Infectious Diseases, Bernhard Nocht Institute for Tropical Medicine, Hamburg, Germany; 3 German Centre for Infection Research, Partner Site Hamburg-Lübeck-Borstel-Riems, Hamburg, Germany; 4 First Department of Medicine, Division of Infectious Diseases, University Medical Centre Hamburg-Eppendorf, Hamburg, Germany; 5 Department of Biomolecular Systems, Max Planck Institute of Colloids and Interfaces, Potsdam, Germany; 6 Institute for Virology, Philipps University Marburg, Marburg, Germany; 7 German Centre for Infection Research, Partner Site Gießen-Marburg-Langen, Marburg, Germany; 8 Faculty of Medicine and University Hospital of Cologne, Department I of Internal Medicine, Centre for Integrated Oncology Aachen Bonn Cologne Düsseldorf (CIO ABCD), German CLL Group (GCLLSG), University of Cologne, Cologne, Germany; 9 German Centre for Infection Research, Partner Site Bonn-Cologne, Cologne, Germany; 10 Clinical Trial Center North GmbH & Co. KG, Hamburg, Germany; 11 German Centre for Infection Research, Translational Project Management Office, Brunswick, Germany; 12 Institute of Virology, University of Veterinary Medicine Hannover, Foundation, Hanover, Germany; 13 German Centre for Infection Research, Partner Site Hannover-Brunswick, Hanover, Germany; 14 Division of Virology, Department of Veterinary Sciences, Institute for Infectious Diseases and Zoonoses, LMU Munich, Munich, Germany; 15 German Centre for Infection Research, Partner Site Munich, Munich, Germany

**Keywords:** Phase I trials, Live attenuated vaccines, Immunological memory, Antibodies, Viral infection

## Abstract

In response to the COVID-19 pandemic, multiple vaccines were developed using platforms such as viral vectors and mRNA technology. Here, we report humoral and cellular immunogenicity data from human phase 1 clinical trials investigating two recombinant Modified Vaccinia virus Ankara vaccine candidates, MVA-SARS-2-S and MVA-SARS-2-ST, encoding the native and the prefusion-stabilized SARS-CoV-2 spike protein, respectively. MVA-SARS-2-ST was more immunogenic than MVA-SARS-2-S, but both were less immunogenic compared to licensed mRNA- and ChAd-based vaccines in SARS-CoV-2 naïve individuals. In heterologous vaccination, previous MVA-SARS-2-S vaccination enhanced T cell functionality and MVA-SARS-2-ST boosted the frequency of T cells and S1-specific IgG levels when used as a third vaccination. While the vaccine candidate containing the prefusion-stabilized spike elicited predominantly S1-specific responses, immunity to the candidate with the native spike was skewed towards S2-specific responses. These data demonstrate how the spike antigen conformation, using the same viral vector, directly affects vaccine immunogenicity in humans.

## Introduction

Severe acute respiratory syndrome coronavirus 2 (SARS-CoV-2) causing coronavirus disease 2019 (COVID-19) has led to significant morbidity and mortality, which was alleviated by the rapid availability of effective vaccines^1–4^. The accelerated development of COVID-19 vaccines was, in part, possible because vaccine platforms such as mRNA and viral vectors were optimized prior to the pandemic and were quickly adjusted to encode a new antigen upon the emergence of SARS-CoV-2^5^.

One promising vaccine platform against emerging viruses is the recombinant Modified Vaccinia virus Ankara (rMVA), an attenuated poxviral vector that efficiently infects, but cannot replicate, in human cells. While non-recombinant MVA is a licensed vaccine against smallpox and monkeypox, the rMVA viral vector platform was recently approved in a heterologous prime-boost regimen against Ebola (Mvabea) and has been investigated in various clinical trials, including an rMVA-based multivalent RSV vaccine currently undergoing phase III efficacy testing^6–8^. Clinical trials using MVA have included immunocompromised patients and infants, providing extensive favorable safety data^9,10^. Using the rMVA platform, two vaccine candidates against COVID-19 were developed^11,12^, leveraging prior experience with an rMVA-based vaccine candidate (MVA-MERS-S) against *Middle East respiratory syndrome* (MERS), which encodes the native, full-length MERS-CoV spike (S)-protein, and was shown to be safe and immunogenic in a first-in-human phase 1 clinical trial^13–15^. The S-protein of *Betacoronaviruses* consists of the S1 subunit binding the host cell receptor and the S2 subunit, which mediates fusion with the cell membrane upon S1/S2 cleavage. Both subunits are important targets for antibodies that can interfere with virus entry, thus making the S-protein a promising vaccine antigen^16–18^.

MVA-SARS-2-S (MVA-S) encodes the native, full-length SARS-CoV-2 S-protein. MVA-SARS-2-ST (MVA-ST) encodes a modified S-protein with two proline amino acid substitutions in the S2 subunit and additional mutations to inactivate the S1/S2 cleavage site. These modifications render the S-protein in a prefusion-stabilized conformation that is not cleaved into S1 and S2 subunits, but anchored on the membrane of MVA-ST-infected cells^12^. Both vaccine candidates showed protective efficacy in mice and hamsters^11,12^ and proceeded to evaluation in phase 1 clinical trials in October 2020 (MVA-S, ClinicalTrials.gov: NCT04569383) and July 2021 (MVA-ST, ClinicalTrials.gov: NCT04895449), respectively (see Supplementary Note 1 for details). MVA-S and MVA-ST were administered to SARS-CoV-2 naïve individuals in a two-dose immunization schedule, 28 days apart. Additionally, MVA-ST was investigated as a one-dose booster vaccination for mRNA-vaccinated individuals.

To comparatively evaluate the immunogenicity of the rMVA-based COVID-19 vaccine candidates, we included the first three COVID-19 vaccines licensed in the EU in the analysis. BNT162b2 (Comirnaty) and mRNA-1273 (Spikevax), here referred to as mRNA, encode a prefusion-stabilized SARS-CoV-2 S-protein with the native S1/S2 cleavage site^19–21^. ChAdOx1 nCov-19 (Vaxzevria), here referred to as ChAd, is a viral vector vaccine based on a replication-deficient chimpanzee adenovirus encoding the native, full-length SARS-CoV-2 S-protein^22,23^. The high efficacy of the mRNA and ChAd vaccines against symptomatic COVID-19 has been associated with high titers of S-specific binding immunoglobulin G (IgG) and neutralizing antibodies^24,25^. However, no distinct correlate of protection has been defined, and the underlying mechanisms leading to and maintaining protection remain elusive. Important parameters, such as memory B and T cells that contribute to long-term immune memory, are often not part of primary analyses^26–29^.

To investigate the immunogenicity of the two rMVA-based vaccine candidates in comparison to the licensed mRNA and ChAd vaccines in humans, peripheral blood samples were collected prior to and at multiple defined time points after vaccination, allowing for a comprehensive and longitudinal comparison of the immune response in individuals receiving five different vaccination regimens. We specifically analyzed S1- and S2-specific antibody isotypes and IgG subclasses, and identified potential IgG epitopes. In addition, we performed a longitudinal analysis of S-specific B cells and analyzed the magnitude and cytokine profile of S-specific T cells. Our findings highlight distinct S1- and S2-specific characteristics of the adaptive immune response induced by the same viral vector platform but encoding different conformations of the S-antigen, which is an important approach to inform future antigen design.

## Results

**Fig. 1:**
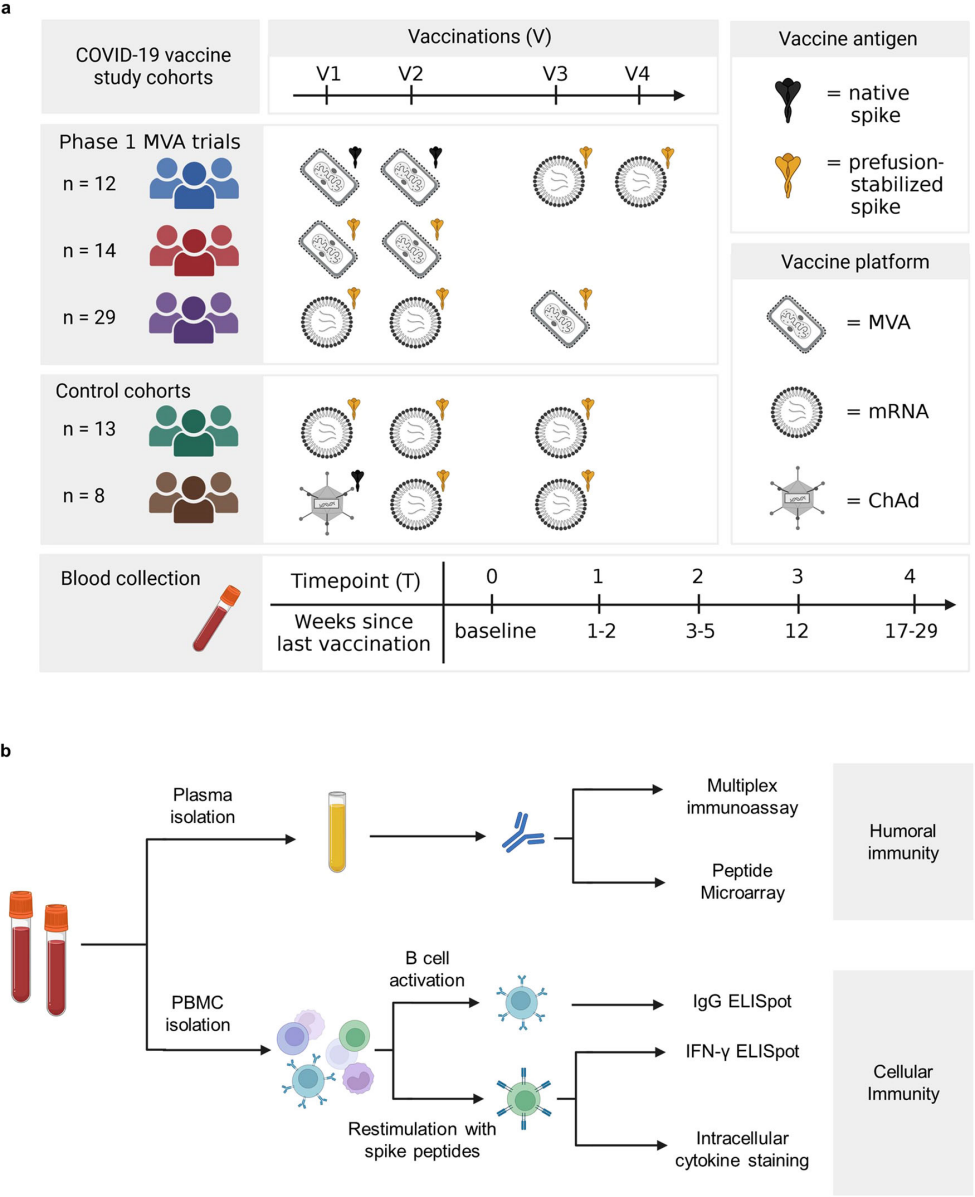
Study design. **a** Participants of five study cohorts received up to four vaccinations (V1 to V4) with different COVID-19 vaccines. The vaccines administered in this study include the two experimental rMVA-based vaccine candidates MVA-SARS-2-S (MVA-S) and MVA-SARS-2-ST (MVA-ST), as well as the licensed vaccines BNT162b2 and mRNA-1273 (together referred to as mRNA) and ChAdOx1 nCov-19 (ChAd). The different vaccines encode either the native spike protein (black) or the prefusion-stabilized spike (yellow). Blood samples were collected at different time points after vaccination, labeled as T0 (baseline), T1 (1–2 weeks), T2 (3–5 weeks), T3 (12 weeks), and T4 (17–29 weeks), referring to the time since last vaccination (V1–V4). **b** The humoral and cellular immune response was analyzed using different assays. See Supplementary Tables 4–8 for detailed number of samples and analyzed time points by assay and study cohort.

To gain insight into the humoral and cellular immune responses induced by two novel rMVA-based COVID-19 vaccine candidates encoding different conformations of the S-protein, we evaluated immunogenicity in three cohorts receiving MVA-S (native S) or MVA-ST (prefusion-stabilized S) in combination with licensed vaccines (MVA-S/mRNA (blue), MVA-ST (red), and mRNA/MVA-ST cohorts (purple)). For comparison, we recruited two control cohorts vaccinated with the licensed ChAd and mRNA vaccines (mRNA (green) and ChAd/mRNA (brown) cohorts). Only participants without SARS-CoV-2 infections before and during the entire study period were included in this analysis (as detailed in the methods section). A detailed description of the vaccine regimens and antigens is shown in Fig. 1a. Participant demographics and time intervals between vaccinations are shown in Supplementary Tables 1 and 2 and Supplementary Fig. 1. Peripheral blood samples were collected longitudinally at T0 (baseline before vaccination), T1 (1–2 weeks), T2 (3–5 weeks), T3 (12 weeks), and T4 (17–29 weeks) post vaccination (Fig. 1a; Supplementary Table 3), with weeks referring to the time since the last vaccination (V1-V4). In total, blood samples were obtained from 76 donors, longitudinally (Supplementary Fig. 1). We longitudinally measured antigen-specific serum antibodies and performed several phenotypic B and T cell assays, as described in Fig. 1b. Sample sizes of each analysis and timepoint, and statistics of the following paragraphs are indicated in Supplementary Tables 4 to 12.

### S1/S2-specific IgG response induced by MVA-S and MVA-ST immunization

**Fig. 2:**
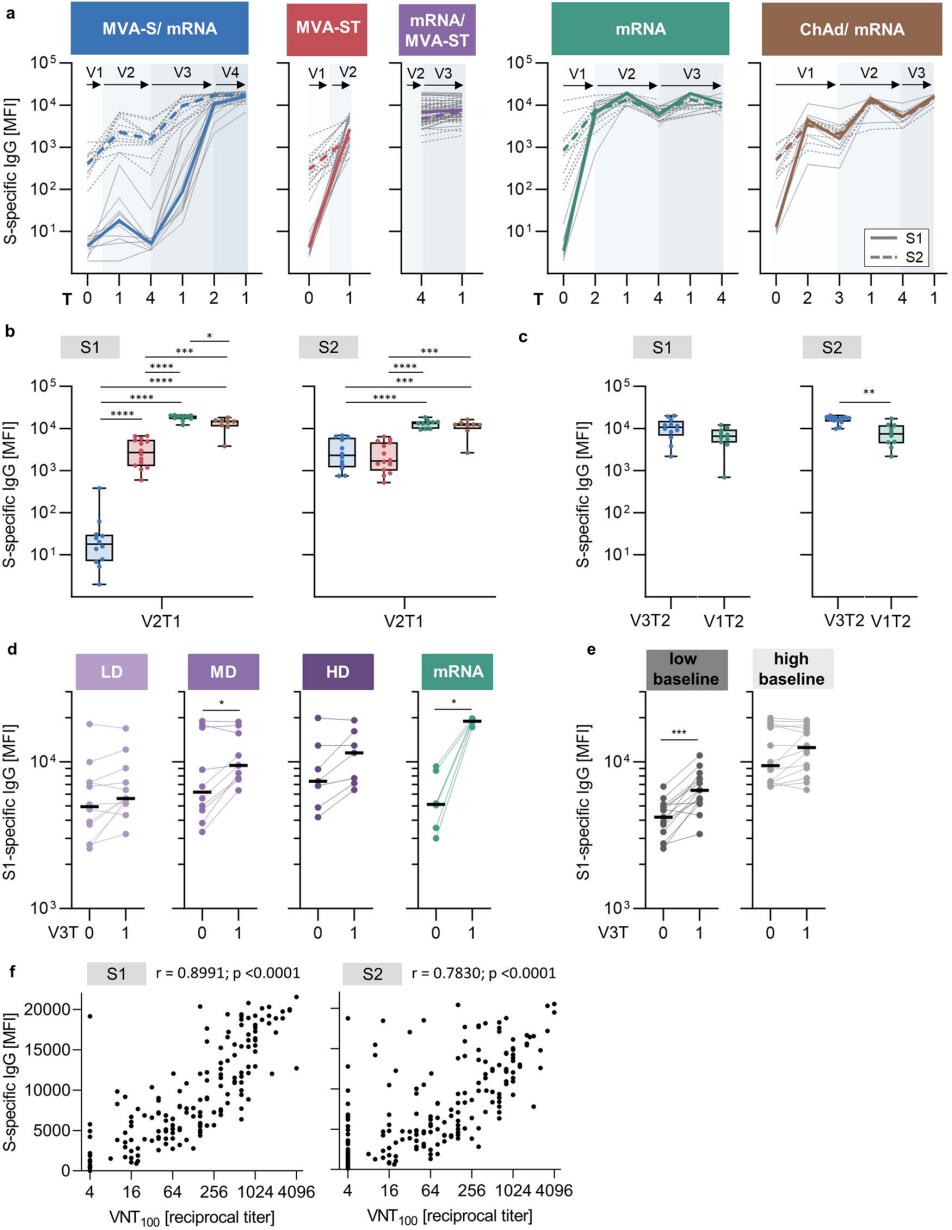
S1/S2-specific IgG response induced by MVA-S and MVA-ST immunization. **a** S1 (continuous line)- and S2 (dashed line)- specific IgG responses measured at baseline and longitudinally after each vaccination. Colored lines depict median MFI (measured by bead-based multiplex immunoassay, mean of technical duplicates). Gray lines show dynamics of individual study participants. Vaccinations V1 to V4 are indicated by arrows. **b** S1- (left) and S2- (right) specific IgG levels induced by two doses of MVA-S (blue) or MVA-ST (red) at V2T1 in comparison to the control cohorts (green and brown). **c** S1- (left) and S2- (right) specific IgG levels after first mRNA vaccination in the MVA-S/mRNA cohort (blue; V3T2) in comparison to the mRNA control cohort (green; V1T2). **d**, **e** S1-specific IgG levels induced by third vaccination with MVA-ST divided into (**d**) dose groups (purple; LD = low dose, MD = middle dose, HD = high dose) compared to mRNA (green) or by (**e**) low (≤median V3T0; left) and high baseline (>median V3T0; right). Data are represented as median ± IQR (**b**, **c**) or individual data points and median (**d**, **e**). **f** Spearman correlation of serum neutralizing capacity (measured by SARS-CoV-2 virus neutralization test, VNT_100_) with S1- (left) and S2- (right) specific IgG, *n* = 228. Significant p-values are indicated as calculated by two-tailed Mann–Whitney *U* test (**b**, **c**) or Wilcoxon matched-pairs signed rank test (**d**, **e**) and adjusted for multiple comparisons using a Benjamini & Hochberg correction: *p < 0.05, **p < 0.01, ***p < 0.001, ****p < 0.0001. Time points after vaccination are indicated as T0 (baseline), T1 (1-2 weeks), T2 (3–5 weeks), T3 (12 weeks), and T4 (17–29 weeks) (**a**, **b**, **c**, **d**, **e**). *P* values and sample sizes are indicated in Supplementary Tables 9 and 4 to 8.

First, plasma antibodies against the S1 and S2 subunits of the S-protein were measured longitudinally using a bead-based multiplex immunoassay to quantify the relative antibody response based on the median fluorescence intensity (MFI). Here, we highlight the S1- and S2-specific IgG responses (Fig. 2). IgM and IgA responses are shown in Supplementary Fig. 2.

The dynamics of the IgG response for the five different cohorts are shown in Fig. 2a. S1-specific IgG (solid line) was undetectable, whereas S2-specific IgG (dashed line) was present at baseline (T0) in all cohorts. Two immunizations using the MVA-S vaccine candidate (MVA-S/mRNA cohort) induced S1-specific IgG (median fold change (mfc) [MFI] = 3.5; *p* = 0.0104) and S2-specific IgG (mfc [MFI] = 8.2; *p* = 0.0012) above baseline. S1-specific IgG waned over a period of six months to baseline levels (mfc [MFI] = 1, ns), whereas S2-specific IgG was maintained significantly above baseline (mfc [MFI] = 4.3; *p* = 0.0030). Following subsequent mRNA vaccination, both S1- and S2-specific IgG were rapidly boosted, and thereafter followed similar dynamics compared to the control cohorts (see green and brown cohorts in Fig. 2a). In comparison, two vaccinations with MVA-ST (MVA-ST cohort) induced S2-specific IgG (mfc [MFI] = 7.2, *p* = 0.0005) with a similar fold-change as MVA-S but led to a more robust induction of S1-specific IgG (mfc [MFI] = 509.9; *p* = 0.0005) compared to baseline. When using MVA-ST as a third vaccination in previously mRNA vaccinated individuals (mRNA/MVA-ST cohort), the fold-induction of S1- and S2-specific IgG levels above baseline before third vaccination was low (S1: mfc [MFI] = 1.2, *p* = 0.0007; S2: mfc [MFI] = 1.2, *p* = 0.0018). In comparison, the fold-induction of IgG levels after third mRNA vaccination was higher and reached similar levels as seen after second vaccination, both in the mRNA cohort (S1: mfc [MFI] = 3.7, *p* = 0.0421; S2: mfc [MFI] = 2.5, *p* = 0.0421) and the ChAd/mRNA cohort (S1: mfc [MFI] = 2.9, ns; S2: mfc [MFI] = 2.8, ns).

To analyze differences in the specificity of the antibody response towards the S1 or S2 subunit, we directly compared the median MFI (mMFI) IgG levels at the time points of the peak response between the different cohorts. Figure 2b depicts the IgG level at V2T1, where MVA-ST-induced significantly higher S1-specific IgG levels (mMFI = 2704) compared to MVA-S (mMFI = 18; *p* < 0.0001), but lower compared to mRNA (mMFI = 19295, p < 0.0001) and ChAd/mRNA (mMFI = 14558, *p* = 0.0006). In contrast, S2-specific IgG was induced at similar levels by MVA-S (mMFI = 2296) and MVA-ST (mMFI = 1697, ns). The S2-specific IgG response after MVA-S vaccination was significantly lower compared to those of the mRNA (mMFI = 13186; *p* < 0.0001) and ChAd/mRNA (mMFI = 12160, *p* = 0.0008) control cohorts. In Fig. 2c, we compared the time point V3T2 of the MVA-S/mRNA cohort to the time point V1T2 of the mRNA control cohort (both corresponding to T2 after the first mRNA vaccination) to determine whether previous MVA-S vaccination had an effect on the IgG response induced by subsequent mRNA vaccination. At this time point, S2-specific IgG was significantly higher in the MVA-S/mRNA compared to the mRNA cohort (MVA-S/mRNA: mMFI = 17071; mRNA: mMFI = 7468, *p* = 0.0014). S1-specific IgG levels of the MVA-S/mRNA cohort (mMFI = 10687) also tended to be higher than those of the mRNA cohort (mMFI = 6590, ns).

Next, we evaluated the IgG response following third vaccination by stratifying the MVA-ST cohort based on vaccine dose (Fig. 2d) and pre-boosting IgG levels (Fig. 2e). Comparable inductions were observed in the middle-dose (mfc [MFI] = 1.6, *p* = 0.0285) and high-dose groups (mfc [MFI] = 1.5, ns), and a lower induction in the low-dose group (mfc [MFI] = 1.2, ns). Notably, there was a significant S1-specific IgG induction in individuals with low baseline levels before MVA-ST booster vaccination (*p* = 0.0006), but not in those with a high baseline, regardless of the MVA-ST dose group (Fig. 2e).

Of the 257 samples analyzed by bead-based immunoassay, a total of 228 samples across all cohorts were additionally analyzed by SARS-CoV-2 virus neutralization test (VNT_100_). Neutralization capacity strongly correlated with the levels of S1-specific IgG (*r* = 0.8991, *p* < 0.0001), and to a lesser extent with S2-specific IgG (*r* = 0.7830, *p* < 0.0001) (Fig. 2f). Correlations of neutralizing capacity with S1- and S2-specific IgG responses are stratified by cohort in Supplementary Fig. 3.

### Dynamics of IgG subclasses and identification of immunogenic S1/S2-specific B cell epitopes

**Fig. 3:**
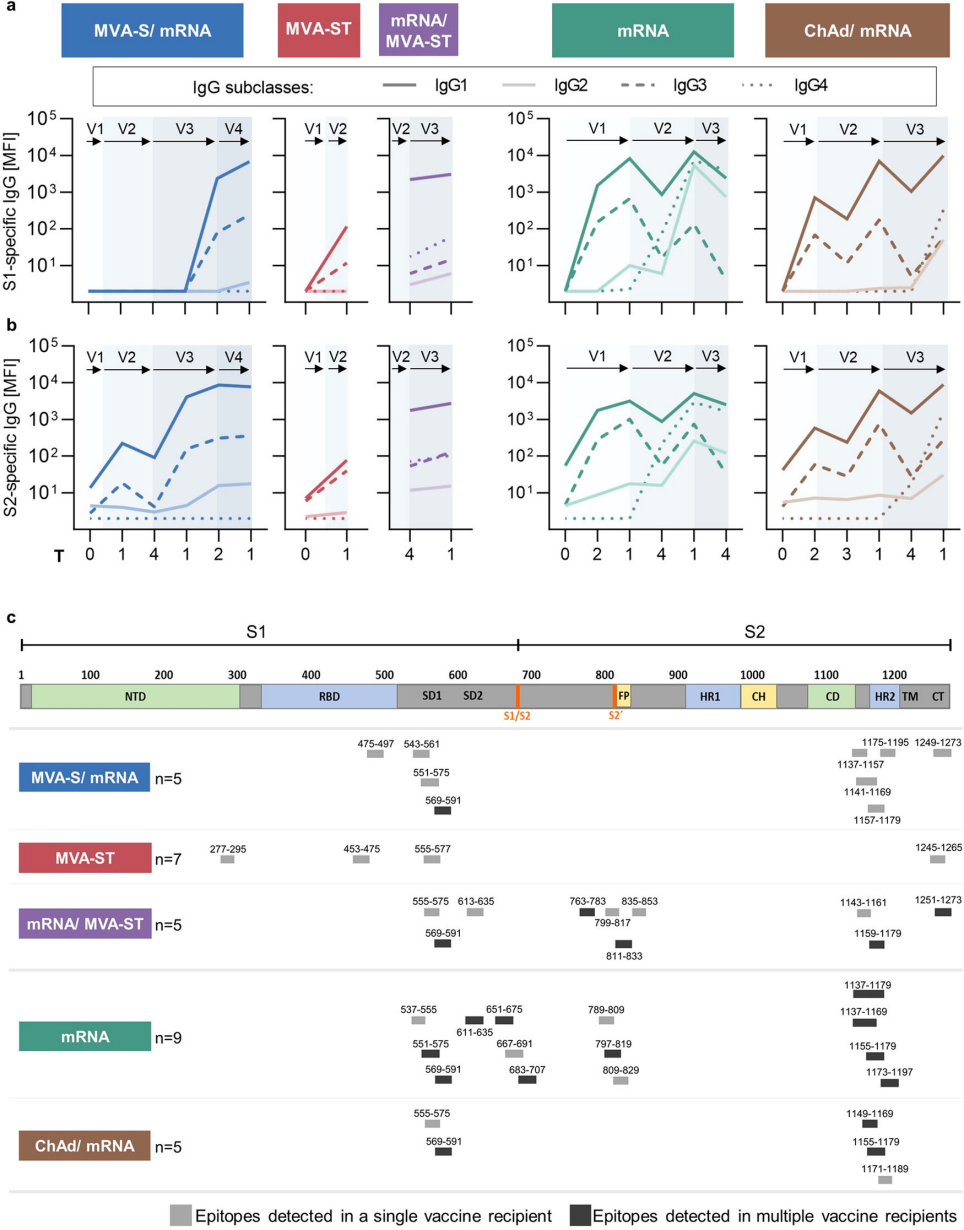
Dynamics of IgG subclasses and identification of immunogenic S1/S2-specific B cell epitopes. **a** S1- (top) and S2- (bottom) specific IgG subclasses of the different study cohorts measured at baseline and longitudinally after each vaccination. Median MFIs (measured by bead-based multiplex immunoassay, mean of technical duplicates) of IgG1-4 are shown as differently dotted lines. Vaccinations V1 to V4 are indicated by arrows and time points after vaccination are indicated as T0 (baseline), T1 (1–2 weeks), T2 (3-5 weeks), T3 (12 weeks), and T4 (17–29 weeks). **b** Schematic representation of immunogenic B cell epitopes measured on peptide microarrays and identified by increased fluorescent intensity (as arbitrary fluorescence units, AFU) in the five study cohorts in one (gray) or multiple (black) individuals, aligned to a schematic depiction of the S-protein^18^. Positive epitope binding was defined as >400 mean AFU of three successive peptides and 2.5-fold above baseline before first vaccination (if available). Time points analyzed after vaccination: mRNA (V2:T1), ChAd/mRNA (V2:T1; V3:T1), MVA-S/mRNA (V2:T1; V4:T1), MVA-ST (V2:T1), mRNA/MVA-ST (V3:T0; V3:T1). (NTD: N-terminal domain, RBD receptor-binding domain, SD1, SD2 subdomain 1 and 2, S1/S2 S1/S2 cleavage site, S2‘ S2’cleavage site, FP fusion peptide, HR1 heptad repeat 1, CH central helix, CD connector domain, HR2 heptad repeat 2, TM transmembrane domain, CT cytoplasmic tail). Sample sizes are indicated in Supplementary Tables 4–8.

We longitudinally analyzed the S1- (top panel) and S2- (bottom panel) specific IgG1-IgG4 responses using the bead-based multiplex immunoassay (Fig. 3a). Primary vaccination with MVA-ST, mRNA, and ChAd mainly induced IgG1 and IgG3, which were boosted by subsequent vaccinations. Overall, both IgG1 and IgG3 followed kinetics similar to those observed for total IgG (Fig. 2a). Notably, the IgG2 and IgG4 subclasses were only induced in the mRNA and ChAd/mRNA control cohorts, where they were first detectable after the second dose of mRNA vaccination, which corresponds to V2 in the mRNA cohort and V3 in the ChAd/mRNA cohort. The IgG2 and IgG4 responses were further boosted after the third mRNA vaccination in the mRNA cohort (Fig. 3a).

To further investigate epitope specificity of the humoral response, we analyzed IgG and IgA binding to S-specific peptides using microarrays. Heatmaps of all cohorts depicting antibody binding measured in arbitrary fluorescent units (AFU) are shown in Supplementary Data 1. Vaccine-induced responses were defined by comparing the AFU after vaccination with the baseline. We did not detect a change in the binding of IgA antibodies to linear S-specific epitopes after vaccination compared to the baseline (Supplementary Data 1). IgG binding to S-specific epitopes was detected in all cohorts after vaccination (Fig. 3b). As shown in Supplementary Data 1, the epitope breadth varies, depending on the grouping of epitopes and the number of participants analyzed per cohort. Immunogenic regions within SARS-CoV-2 S, in which vaccine recipients from several cohorts showed antibody binding, were identified in the S1 subunit (amino acids (AA) 537-635) and in the S2 subunit (AA 763-853, AA 1137-1159). One region in the S1/S2 junction (AA 651-707) was identified only in the mRNA cohort. Notably, epitopes in the cytoplasmic tail of the S2 subunit (AA 1245-1273) were detected only in the cohorts that had received an MVA-based vaccination.

### Longitudinal analysis of S1/S2-specific B cell responses induced by MVA-S and MVA-ST immunization

**Fig. 4:**
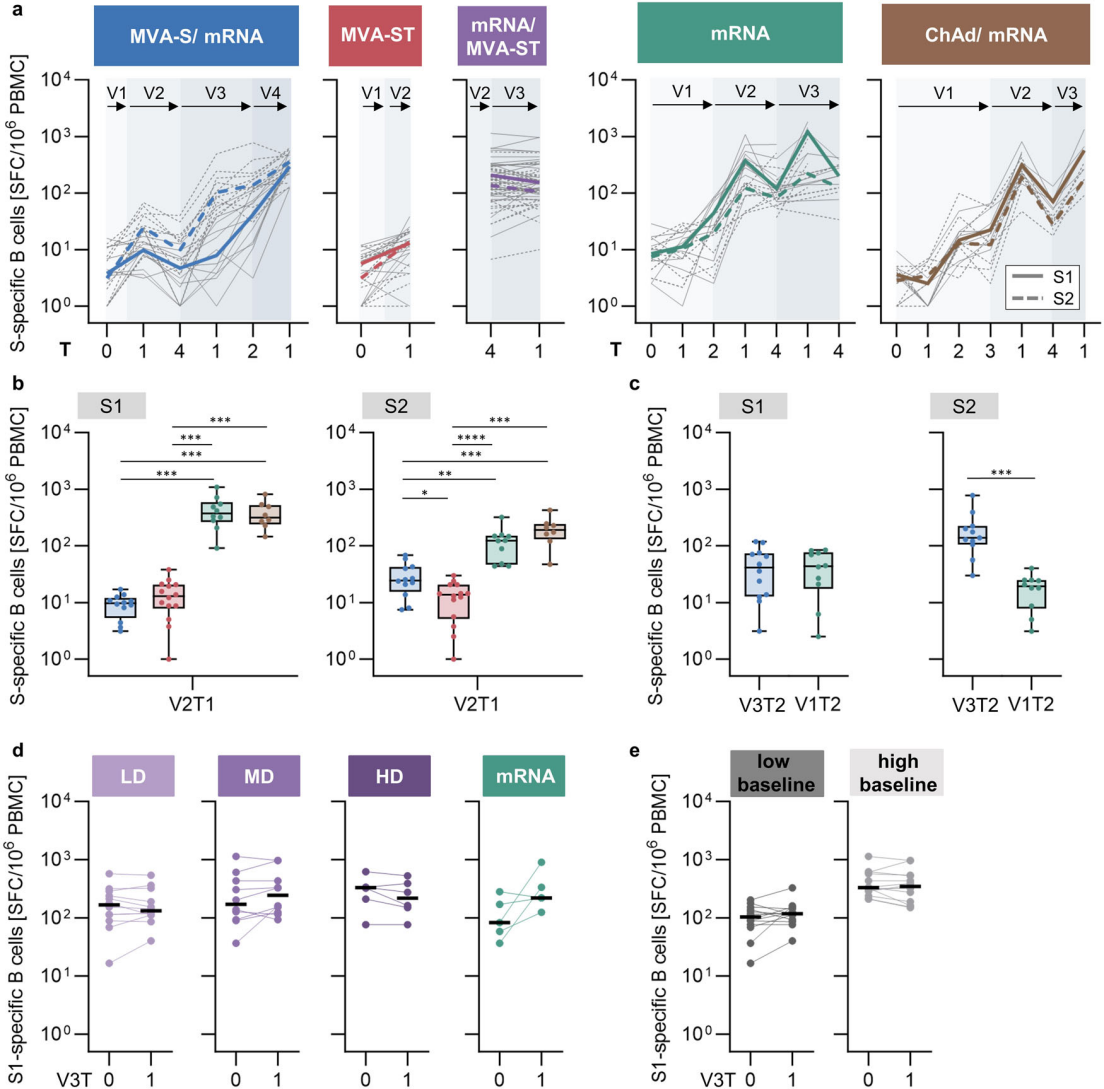
Longitudinal analysis of S1/S2-specific B cell responses induced by MVA-S and MVA-ST immunization. **a** Frequencies of IgG-secreting B cells shown as SFC/10^6^ PBMCs (mean of technical duplicates) measured by IgG ELISpot. Colored lines depict median S1- (continuous line) and S2- (dashed line) specific responses for each cohort. Gray lines show the dynamics of individual participants. **b** S1- (left) and S2- (right) specific IgG-secreting B cells induced by two doses MVA-S (blue) or MVA-ST (red) at V2T1 in comparison to the control cohorts (green and brown). **c** S1- (left) and S2- (right) specific IgG-secreting B cells after first mRNA vaccination in the MVA-S/mRNA cohort (blue; V3T2) in comparison to first vaccination in the mRNA control cohort (green; V1T2). S1-specific IgG-secreting B cells induced by third vaccination with MVA-ST divided into (**d**) dose groups (purple; LD = low dose, MD = middle dose, HD = high dose) compared to mRNA (green) or by (**e**) low (<median V3T0; left) and high baseline (>median V3T0; right). Data are represented as median ± IQR (**b**, **c**) or individual data points and median (**d**, **e**). Significant *p* values are indicated as calculated by two-tailed Mann–Whitney-U test (**b**, **c**) or Wilcoxon matched-pairs signed rank test (**d**, **e**) and adjusted for multiple comparisons using a Benjamini & Hochberg correction: *p < 0.05, **p < 0.01, ***p < 0.001, ****p < 0.0001 (**b**, **c**). Time points after vaccination are indicated as T0 (baseline), T1 (1–2 weeks), T2 (3–5 weeks), T3 (12 weeks), and T4 (17–29 weeks) (**a**, **b**, **c**). *P* values and sample sizes are indicated in Supplementary Tables 10 and 4–8.

To evaluate whether the observed S1/S2 bias within the antibody response (see Fig. 2) was also reflected in the cellular response, we characterized the B cellular immune response using an antigen-specific IgG enzyme-linked immunospot (ELISpot) assay (Fig. 4). B cells specific for the S1 and S2 subunits were quantified as IgG spot-forming cells (SFC) per million peripheral blood mononuclear cells (PBMCs), and total IgG-secreting B cells served as a positive control.

In the MVA-S/mRNA cohort, a slight induction of S1-specific B cells (S1: mfc [SFC] = 1.5, *p* = 0.0298) and a more pronounced induction of S2-specific B cells (S2: mfc [SFC] = 7.8, *p* = 0.0018) above baseline levels were observed after two MVA-S vaccinations (V2T1) (Fig. 4a). This observation was consistent with the IgG response (see Fig. 2a). Notably, S2-specific B cells expanded more rapidly after the first mRNA vaccination in this cohort compared to the mRNA control cohort, while S1-specific B cells followed similar dynamics. Vaccination with two doses of MVA-ST induced S1- and S2-specific B cells significantly above baseline, but both at low levels (S1: mfc [SFC] = 2.2, *p* = 0.0156; S2: mfc [SFC] = 2.5, *p* = 0.0078). The fold induction of S1- and S2-specific B cells was also low after the first immunization using mRNA (S1: mfc [SFC] = 5.4, *p* = 0.0201; S2: mfc [SFC] = 3.2, p = 0.0419) or ChAd (S1: mfc [SFC] = 3.5, *p* = 0.0170; S2: mfc [SFC] = 7.2, *p* = 0.0419), but was further increased after the second dose in the mRNA (S1: mfc [SFC] = 33.1, *p* = 0.0066; S2: mfc [SFC] = 13.1, *p* = 0.0156) and ChAd/mRNA (S1: mfc [SFC] = 115.8, *p* = 0.0170; S2: mfc [SFC] = 82.5, *p* = 0.0170) control cohorts. The frequency of S1-and S2-specific B cells did not increase following MVA-ST as a third vaccination (mRNA/MVA-ST cohort) (S1: mfc [SFC] = 1; S2: mfc [SFC] = 1). In contrast, a third mRNA vaccination boosted the B cell response in the mRNA (S1: mfc [SFC] = 8.8; S2: mfc [SFC] = 5.8) and ChAd/mRNA (S1: mfc [SFC] = 5.0; S2: mfc [SFC] = 7.3) control cohorts, but this did not reach statistical significance.

After the primary vaccination series at V2T1, an S1/S2 bias was observed for B cells (Fig. 4b), similar to that detected for IgG (Fig. 2b). S1-specific B cell frequencies were higher, although not significantly, after MVA-ST (median = 13 SFC) than after MVA-S vaccination (median = 10 SFC), but significantly lower than in the mRNA (median = 371 SFC, p = 0.0004) and ChAd/mRNA (median = 316 SFC, *p* < 0.0008) control cohorts. In contrast, S2-specific B cells were induced at significantly higher frequencies by MVA-S (median = 25 SFC) compared to MVA-ST (median = 14 SFC, *p* = 0.0228). The S2-specific B cell response after MVA-S vaccination was still significantly lower than that in the mRNA (median = 122 SFC, *p* = 0.0017) and ChAd/mRNA (median = 191 SFC, *p* = 0.0005) control cohorts. Looking at the effect of primary MVA-S vaccination on subsequent mRNA vaccination, the induction of S2-specific B cells was significantly higher in the MVA-S/mRNA cohort (V3T2: median = 138 SFC) compared to primary vaccination in the mRNA cohort (V1T2: median = 19 SFC, *p* = 0.0008) (Fig. 4c), whereas S1-specific responses did not show a significant difference. We also evaluated the S1-specific B cell response following third MVA-ST vaccination stratified by dose (Fig. 4d) and pre-boosting B cell levels (Fig. 4e). In contrast to the IgG response, no significant induction was observed regardless of the dose group and baseline levels.

### Longitudinal analysis of S1/S2-specific T cell responses induced by MVA-S and MVA-ST immunization

**Fig. 5:**
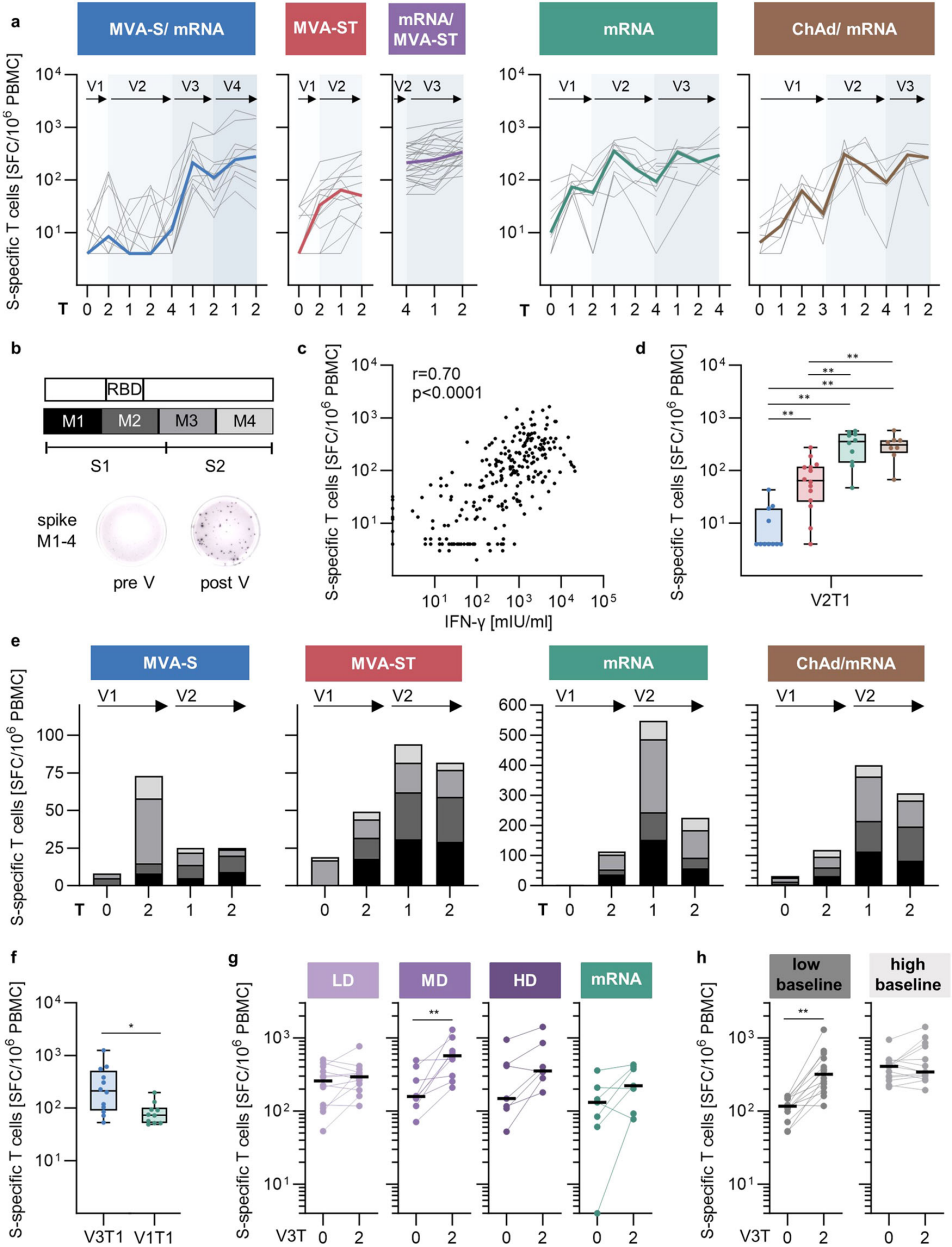
Longitudinal analysis of S1/S2-specific T cell responses induced by MVA-S and MVA-ST immunization. **a** Frequencies of IFN-γ-producing T cells (SFC/10^6^ PBMCs; mean of technical triplicates) measured by ELISpot. Colored lines depict median responses, gray lines show the dynamics of individual participants. **b** Schematic of the peptide pools M1-4 resembling the spike S1 and S2. Representative ELISpot wells pre (left) and post vaccination (right)**. c** Spearman correlation of ELISpot and IFN-γ release assay. T cell response induced by two doses MVA-S (blue) or MVA-ST (red) at (**d**) V2T1 or (**e**) against individual peptide pools M1-M4 compared to the control cohorts (green and brown). **f** T cell responses after first mRNA vaccination in the MVA-S/mRNA cohort (blue; V3T1) compared to the mRNA control cohort (green; V1T1). T cell responses induced by third vaccination with MVA-ST divided into (**g**) dose groups (purple; LD = low dose, MD = middle dose, HD = high dose) compared to mRNA (green) or by (**h**) low ( ≤ median V3T0; left) and high baseline (>median V3T0; right). Data are represented as median ± IQR (**d**, **f**), sum of medians (**e**), or individual data points and median (**g**, **h**). Significant *p* values are indicated as calculated by two-tailed Mann–Whitney *U* test (**d**, **f**) or Wilcoxon matched-pairs signed rank test (**g**, **h**) and adjusted for multiple comparisons using a Benjamini & Hochberg correction: *p < 0.05, **p < 0.01, ***p < 0.001, ****p < 0.0001. Time points after vaccination are indicated as T0 (baseline), T1 (1–2 weeks), T2 (3–5 weeks), T3 (12 weeks), and T4 (17–29 weeks) (**a**, **d**, **e**, **f**, **g**, **h**). *P* values and sample sizes are indicated in Supplementary Tables 11 and 4–8.

It is strongly suggested that T cells contribute significantly to vaccine-induced immunity and protection. Here, the S-specific T cell dynamics following vaccination were analyzed by IFN-γ ELISpot assay, as shown in Fig. 5a for the different cohorts. PBMCs were stimulated with an overlapping peptide (OLP) pool consisting of four individual pools (M1-M4) spanning the entire SARS-CoV-2 S-protein (Fig. 5b), with M1–M2 corresponding predominantly to S1 and M3-M4 to S2. The results were quantified as IFN-γ-secreting SFC per million PBMCs. Representative ELISpot wells are shown in Fig. 5b. To confirm the T cell results in an independent assay, we used a commercial whole-blood IFN-γ release T cell assay. The results correlated with those of the IFN-γ ELISpot assay (Spearman *r* = 0.7; *p* < 0.0001), providing additional evidence for the robustness of the methods (Fig. 5c).

Although MVA-S induced a detectable T cell response in some participants, the response at the peak time point (V1T2) was not significantly higher than that at baseline (mfc [SFC] = 1, ns) (Fig. 5a). Upon subsequent mRNA vaccination in this cohort, S-specific T cell frequencies were boosted 22-fold above baseline, as early as one week after the first mRNA vaccination. In contrast to MVA-S, one dose of MVA-ST induced a T cell response significantly above baseline (mfc [SFC] = 2.4, *p* = 0.0249), which was comparable to one dose of mRNA (mfc [SFC] = 2.9) but lower than that of ChAd (mfc [SFC] = 9.7). When used as a third vaccination in previously mRNA-vaccinated individuals, MVA-ST significantly boosted the T cell response above the baseline (mfc [SFC] = 1.7, *p* = 0.0012). These results are comparable to a third mRNA vaccination where the T cell response was boosted (mfc [SFC] = 2.0, ns) but not above the peak levels seen after the second vaccination.

Peak responses at V2T1 were directly compared between the different cohorts, revealing that MVA-ST induced a significantly higher T cell response than MVA-S (MVA-ST = 65 SFC; MVA-S = 4 SFC, *p* = 0.0021) (Fig. 5d). However, this response was significantly lower compared to the control cohorts (mRNA = 356 SFC, *p* = 0.0021; ChAd/mRNA = 306 SFC, *p* = 0.0023). We then analyzed the T cell response to the two S subunits by evaluating the response to the four OLP pools separately. The pools M1-M2 mainly cover the S1 and M3-M4 the S2 subunit. MVA-S induced a T cell response that was biased towards the S2 subunit after first vaccination. This bias was not observed after the second MVA-S vaccination, where the response was overall low. MVA-ST induced an S1-biased response, that was most pronounced after the second MVA-ST vaccination (Fig. 5e). The same pattern was reflected in the IgG and B cell responses, suggesting a dependence on S-protein conformation. S1- and S2-specific T cells were induced at comparable frequencies in the control cohorts.

To evaluate whether previous MVA-S vaccination affected the resulting T cell response, we directly compared S-specific T cell frequencies at the peak time point after the first mRNA vaccination in the MVA-S/mRNA (V3T1) and mRNA (V1T1) cohorts (Fig. 5f). The IFN-γ T cell response was significantly higher in the MVA-S/mRNA cohort (MVA-S/mRNA = 213 SFC) than in the mRNA control cohort (mRNA = 74 SFC, *p* = 0.0211). When using MVA-ST as a third vaccination, a significant increase above baseline before third vaccination was only seen in the middle-dose group (*p* = 0.0093), with a higher fold-change compared to the other dose groups (LD: mfc [SFC] = 1.1; MD: mfc [SFC] = 2.7; HD: mfc [SFC] = 2.0) (Fig. 5g). Notably, the ability of MVA-ST to boost the T cell response was dependent on the baseline levels before the third vaccination, similar to the IgG response shown in Fig. 2e. A significant induction of the T cell response was only observed in participants with low baseline levels (*p* = 0.0012), in contrast to those with a high baseline (Fig. 5h).

### Enhanced T cell polyfunctionality after previous MVA-S immunization

**Fig. 6:**
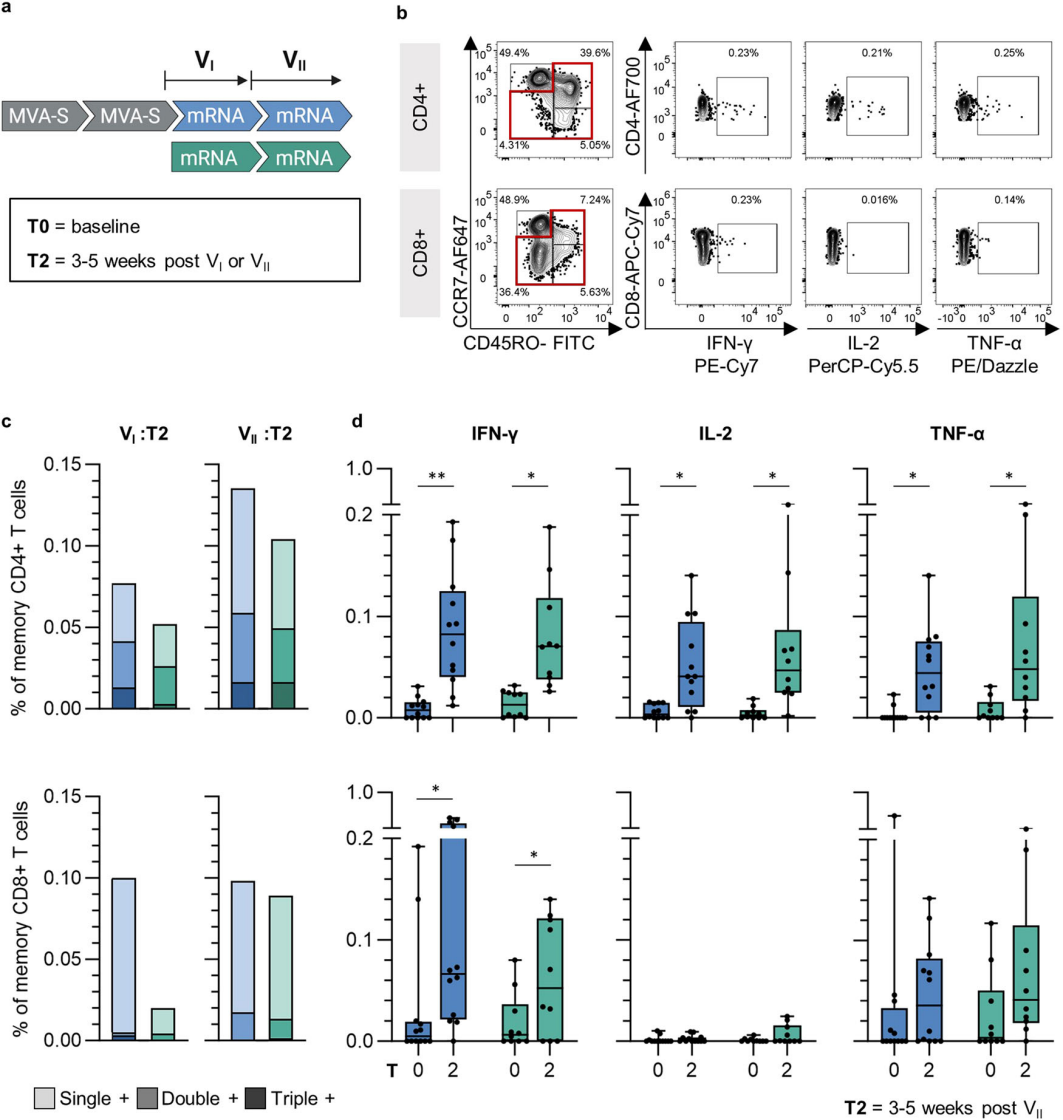
Enhanced T cell polyfunctionality after previous MVA-S immunization. **a** Analyzed study cohorts and time points: MVA-S/mRNA (blue) and mRNA (green) were analyzed at baseline (T0) and time points after first and second vaccination with licensed mRNA vaccines, referred to as V_I_ and V_II_ (T2). **b** Representative gating strategy of cytokine-secreting CD4^+^ (top) and CD8^+^ (bottom) memory T cells after stimulation with overlapping peptide pools covering the S-protein. **c** Median frequencies of single positive (IFN-γ^+^ or IL-2^+^ or TNF-α^+^), double positive (IFN-γ^+^ IL-2^+^ TNF-α^-^ or IFN-γ^+^ IL-2^-^ TNF-α^+^ or IFN-γ^-^ IL-2^+^ TNF-α^+^), and triple positive (IFN-γ^+^ IL-2^+^ TNF-α^+^) T cells out of total CD4^+^ (top) and CD8^+^ (bottom) memory T cells. Results were obtained by Boolean gating of the cytokine gates shown in (**b**). **d** Frequencies of IFN-γ, IL-2, and TNF-α-positive T cells out of total CD4^+^ (top) and CD8^+^ (bottom) memory T cells at baseline and time point T2 post V_I_ and V_II_. Data are represented as individual data points and median ± IQR. Significant *p* values are indicated as calculated by two-tailed Wilcoxon matched-pairs signed rank test and adjusted for multiple comparisons using a Benjamini & Hochberg correction: *p < 0.05, **p < 0.01, ***p < 0.001, ****p < 0.0001. *P* values and sample sizes are indicated in Supplementary Tables 12 and 4–8.

The polyfunctionality of S-specific memory T cells was assessed 3-5 weeks after two-dose mRNA vaccination, corresponding to V3 and V4 in the MVA-S/mRNA cohort and V1 and V2 in the mRNA cohort, respectively (Fig. 6a). Intracellular cytokine staining was used to analyze the production of IFN-γ, interleukin-2 (IL-2) and tumor necrosis factor alpha (TNF-α) by CD4^+^ and CD8^+^ T cells (gating is shown in Fig. 6b and Supplementary Fig. 4).

The median frequency (mfr) of total cytokine-producing CD4^+^ T cells was highest in the MVA-S/mRNA cohort, both after the first and second mRNA vaccinations (Fig. 6c). The mfr of polyfunctional CD4^+^ memory T cells expressing all three cytokines was also higher in the MVA-S/mRNA cohort than the mRNA cohort after the first mRNA vaccination (MVA-S/mRNA: mfr = 0.013%; mRNA: mfr = 0.003%) and comparable between both cohorts post second mRNA vaccination (MVA-S/mRNA: mfr = 0.017%; mRNA: mfr = 0.017%). IFN-γ, IL-2- and TNF-α-producing CD4^+^ memory T cells were significantly above baseline in both cohorts (Fig. 6d).

The S-specific cytokine-producing CD8^+^ memory T cell response was less pronounced than the CD4^+^ response. The memory CD8^+^ T cell response was dominated by cells expressing a single cytokine, with a low frequency of polyfunctional CD8^+^ cells in both cohorts (Fig. 6c). Notably, a higher mfr of total cytokine-producing CD8^+^ memory T cells was observed already after the first mRNA vaccination in the MVA-S/mRNA cohort (mfr = 0.1%) compared to the mRNA cohort (mfr = 0.02%). A significant induction of IFN-γ-producing CD8^+^ memory T cells was observed in the MVA-S/mRNA and mRNA cohorts (Fig. 6d).

Overall, our longitudinal analyses indicated that MVA-ST, which encodes the prefusion-stabilized S, is more immunogenic than MVA-S, which encodes the native S, in SARS-CoV-2 naïve individuals. Both vaccine candidates are less immunogenic than the licensed ChAd and mRNA vaccines. Detailed measurement of humoral and cellular immune parameters revealed a bias towards the S2 subunit after MVA-S vaccination, in contrast to a bias towards the S1 subunit after MVA-ST vaccination, which is reflected in the IgG, B cell, and T cell responses. Despite the lower immunogenicity of MVA-S alone, it showed a recall response of the humoral response and polyfunctional T cells upon subsequent mRNA vaccination.

## Discussion

The recent COVID-19 pandemic has led to unprecedented global efforts toward vaccine development. Several vaccine candidates have been licensed, and a multitude of vaccine candidates are still in different stages of development. Here, we report a comparative study of two rMVA-based vaccine candidates that were tested as primary immunization series and as a booster vaccination in consecutive phase 1 first-in-human clinical trials. While the investigated vaccine candidates share identical rMVA viral vector backbones, MVA-S encodes the native S-protein, and MVA-ST was optimized to encode for a prefusion-stabilized S-protein. Our comparative analysis of MVA-S and MVA-ST provided the opportunity to directly investigate the impact of different S-protein conformations on the vaccine-induced immune response in humans. Two key distinctions were observed: 1) MVA-ST was shown to be more immunogenic than MVA-S, but less immunogenic compared to licensed vaccines, and 2) a differential skewing of the humoral and cellular immune response towards the S1 and S2 subunits after rMVA-based vaccination was observed.

MVA-S and MVA-ST vaccination induced detectable S-specific immune responses, including IgG, B, and T cells, in seronegative individuals, but MVA-S was overall less immunogenic. An mRNA booster dose following two MVA-S vaccinations led to an early induction of S2-specific IgG and B cells, IFN-γ-producing T cells and an overall higher frequency of polyfunctional T cells compared to the control cohort only vaccinated with mRNA. This observation may indicate a priming effect of MVA-S, resulting in a recall response of immune memory upon mRNA vaccination despite the low immunogenicity of MVA-S when administered alone. To date, clinical data from another MVA-based COVID-19 vaccine candidate have been published by Routhu et al. Their synthetic MVA-based vaccine candidate encoding a prefusion-stabilized S-protein similar to our MVA-ST, in combination with the nucleocapsid antigen, was tested as a prime-boost schedule in a phase 1 clinical trial^30–32^. Since different assays were used, immunogenicity cannot be compared directly to our studies. However, the early induction of a T cell response after the first dose and the subsequent induction of the humoral response after the second dose showed similar dynamics to those observed for our MVA-ST vaccine.

Analysis of the specificity of the immune response induced by MVA-S and MVA-ST revealed a differential bias towards the S1 and S2 subunits of the S-protein: Both MVA-S and MVA-ST increased the S2-specific IgG levels that were already detectable at baseline, possibly as a result of cross-reactive antibodies and memory cells from previous infections with common cold coronaviruses, as has been suggested previously^33–35^. In contrast, S1-specific IgG was elicited at significantly higher levels following MVA-ST vaccination. In line with this, the T cell response was also biased towards the S2 subunit by MVA-S and towards the S1 subunit by MVA-ST vaccination. A similar pattern was observed in preclinical rodent studies. Even though S-specific seroconversion was reached in all mice regardless of MVA-S or MVA-ST vaccination, significantly lower S1-specific IgG titers were observed in the MVA-S-vaccinated group, whereas S2-specific titers were comparable^12^. These results are likely explained by the differential cell surface expression of native and prefusion-stabilized S-proteins, as shown in in vitro experiments^12^. Across all cohorts, serum neutralization capacity showed a stronger correlation with S1- compared to S2-specific IgG, in line with the previous finding that the receptor-binding domain (RBD)-containing S1 subunit is the main target of SARS-CoV-2 neutralizing antibodies^36^.

Based on this bias towards the S1 or S2 subunit, which is especially prominent in the IgG response, we studied the vaccine-induced IgG subclasses and epitope specificity in more detail. We observed induction of the pro-inflammatory and highly functional IgG1 and IgG3 subclasses against S1 and S2 in all cohorts, irrespective of vaccination scheme, which is in line with the results of licensed mRNA and ChAd vaccines and our own previous data for MVA-MERS-S^14,37–39^. In contrast, IgG2 and IgG4, both generally associated with low functional potency, were detectable only after the second mRNA dose in our control cohorts and not in the MVA cohorts. Similar observations were recently reported by Irrgang et al.^40^ The prolonged germinal center reaction described after mRNA vaccination may result in a continuous class switch recombination towards anti-inflammatory IgG4^41,42^. However, the clinical relevance of this phenomenon remains poorly understood and requires further investigation. We detected linear IgG epitopes that were predominantly localized outside of the RBD in the C-terminal part of the S1 subunit and along the S2 subunit across all study cohorts. Half of the identified immunogenic regions were recognized by more than one vaccine recipient. Notably, we identified an immunogenic region in the S2 domain (AA 763-853) that contains the epitope specificity (AA 814-826) of a recently described neutralizing antibody with pan-coronavirus reactivity^43^. We also revealed one immunogenic region in the C-terminal transmembrane domain of S2 (AA 1245-1273), which was only detected in the cohorts that had received at least one MVA vaccination. While the S1 subunit contains the RBD and has been shown to be the main target of SARS-CoV-2 neutralizing antibodies, antibodies targeting epitopes in the more conserved S2 subunit may contribute to the protection against SARS-CoV-2 variants and other human coronaviruses, especially if pre-existing cellular memory is present^35,36,44^. Whether the induction of functional IgG responses against immunogenic regions in the S2 subunit provides targets for pan-coronavirus vaccines needs to be evaluated in future studies.

In a comparative analysis with the mRNA and ChAd vaccines, our data suggest that neither rMVA-based candidate reached the immunogenicity elicited by these licensed vaccines. This is particularly interesting for the comparison of the viral vector-based vaccines as they show similar immunogenicity in pre-clinical models^45^. It could be hypothesized that the higher dose of ChAd (5 × 10^10^ viral particles) but also the differential molecular mechanisms of the viral vectors may explain this differential immunogenicity. The MVA genome encodes for several immunomodulatory genes that inhibit innate immune pathways, which may dampen the adaptive immune response^46,47^. Combining viral vectors with mRNA-based vaccines in a heterologous prime-boost schedule may be advantageous in humans^22^. Indeed, Barros-Martins et al. showed that individuals who received a heterologous ChAd/mRNA vaccination compared to a homologous ChAd/ChAd schedule showed stronger antibody responses^48^. This may also apply to combining MVA-ST with an mRNA boost. Notably, the T cell response elicited by a two-dose MVA-ST vaccination was comparable in magnitude to that elicited by one dose of a ChAd or mRNA vaccine. MVA-ST also induced similar T cell frequencies to mRNA when used as a third vaccination. The ability of the MVA platform to efficiently induce T cell responses has been demonstrated for other rMVA-based vaccines^49,50^. Recent follow-up data from our MVA-MERS-S trial demonstrated that a third immunization up to 12 months after the primary vaccination series could enhance the magnitude and persistence of spike-specific antibodies and memory B cells^14,15^. Since the residual S-specific immune response from the two prior mRNA vaccinations negatively impacted the boosting capacity, regardless of the platform used, we were unable to measure the same late booster effect on the humoral response as seen in the MVA-MERS-S trial. The phenomenon of baseline dependency has been reported in observational studies of booster vaccinations using licensed vaccines against several pathogens^51^. Together, these data support the potential of using rMVA-based vaccines as booster vaccinations but also highlight the importance of an optimized time interval between immunizations and the advantage of heterologous vaccination schedules^14,15,23^.

The longitudinal blood sampling of each study participant represents a key strength of our study. However, the frequent blood sampling also limited the blood volume per sampling time point which made further analyses, such as deconvoluting the T cell response, not feasible. Another limitation of our study is the small size of some of the cohorts, resulting in a limited power of statistical tests. Nonetheless, the data reported in our manuscript provide a detailed longitudinal investigation of different vaccine regimens, yielding important insights into the impact of the platform, schedule, and antigen conformation on vaccine-induced immune responses. We showed that the immunogenicity of rMVA-based COVID-19 vaccine candidates in humans can be enhanced by conformational changes in the S-protein. Studies such as the one presented here add to a more comprehensive understanding of the strengths and limitations of the rMVA vector technology in comparison to other vaccine platforms. However, critical knowledge gaps, such as the correlates of protection for rMVA-based vaccines, remain to be addressed. A phase 1 clinical study investigating MVA-ST administered by inhalation (ClinicalTrials.gov: NCT05226390) is currently ongoing and may yield critical insights into the safety and immunogenicity of MVA targeting the respiratory mucosal layer^52^.

## Methods

### Vaccines

MVA-SARS-2-S (MVA-S) is a vaccine candidate based on rMVA, encoding the native full-length S-protein of SARS-CoV-2^11^. MVA-SARS-2-ST (MVA-ST) is an optimized version of MVA-S that encodes a pre-fusion-stabilized S protein with an inactivated S1/S2 furin cleavage site, as described by Natrup et al. ^12^. BNT162b2 (Comirnaty) and mRNA-1273 (Spikevax), here referred to as mRNA, are licensed vaccines consisting of nucleoside-modified mRNA encoding the prefusion-stabilized S-protein, formulated in lipid-nanoparticles^1,4^. ChAdOx1 nCoV-19 Vaxzevria, herein referred to as ChAd, is a licensed vaccine based on the modified chimpanzee adenovirus ChAdOx1 vector, encoding the full-length S-protein and a tissue plasminogen activator leader sequence^22^.

### Study approval

The following phase 1 clinical trials were reviewed and approved by the National Competent Authority (Paul-Ehrlich-Institute, EudraCT numbers 2020-003875-16; 2021-000548-23) and the Ethics Committee of the Hamburg Medical Association (reference numbers 2020-10164-AMG-ff; 2021-100621-AMG-ff), conducted under the sponsorship of the University Medical Center Hamburg-Eppendorf (Hamburg, Germany) in accordance with ICH-GCP and the EU directives 2001/20/EC and 2001/83/EC, and are registered at ClinicalTrials.gov. (NCT04569383; NCT04895449). The Ethics Committee of the Hamburg Medical Association approved the clinical study with licensed vaccines (reference number: 2020-10376-BO-ff). Written informed consent was obtained from all participants.

### Study design

NCT04569383 is a phase 1 clinical trial to evaluate the MVA-SARS-2-S vaccine candidate in 30 seronegative individuals divided into two ascending dose groups. Participants received two single injections 28 days apart, either a low dose of 1 × 10^7^ ± 0.5 log IU (N = 15) or a high dose of 1 × 10^8^ ± 0.5 log IU (*N* = 15). The MVA-S/mRNA cohort is a subgroup of this trial (*N* = 12), which received two doses of the BNT162b2 vaccine 21 days apart, at least six months after the last MVA-SARS-2-S vaccination.

NCT04895449 is a phase 1b clinical trial to evaluate the MVA-SARS-2-ST vaccine candidate in seronegative individuals (Part A) and in individuals who had previously received two doses of the BNT162b2 vaccine (Part B). In Part A, participants received two single injections 28 days apart, either a low dose of 1 × 10^7^ ± 0.5 log IU (*N* = 8) or a middle dose of 5 × 10^7^ ± 0.5 log IU (*N* = 7). In Part B, participants received a single injection of low dose 1 × 10^7^ ± 0.5 log IU (*N* = 12), middle dose 5 × 10^7^ ± 0.5 log IU (*N* = 10), or high dose 1 × 10^8^ ± 0.5 log IU (*N* = 8) MVA-SARS-2-ST at least six months after their last BNT162b2 vaccination. Here, the MVA-ST cohort refers to Part A, whereas the mRNA/MVA-ST cohort refers to Part B of this study (Fig. 1A).

The mRNA and ChAd/mRNA study cohorts consisted of participants who received two doses of mRNA vaccine (21 or 28 days apart) or one dose ChAd plus one dose mRNA (84 days apart), respectively, and a booster vaccination of mRNA after six months. The studies were conducted at the University Medical Center Hamburg-Eppendorf.

### Exclusion criteria

For the phase 1 clinical trials testing the MVA-based vaccine candidates (MVA-S/mRNA; MVA-ST; mRNA/MVA-ST cohorts), SARS-CoV-2 exposure prior to the study was an exclusion criterion. Individuals with a positive SARS-CoV-2 PCR in medical history (MVA-S/mRNA; MVA-ST; mRNA/MVA-ST cohorts) and/or positive SARS-CoV-2 antibody test at screening day (MVA-S/mRNA; MVA-ST cohorts) were not included in the clinical trials. Additionally, active SARS-CoV-2 infection on screening day was excluded by PCR (MVA-S/mRNA) or antigen test followed by PCR if positive (MVA-ST; mRNA/MVA-ST). Participants of these phase 1 clinical trials were instructed to report clinical evidence of COVID-19-like symptoms to the study site per study protocol. In these cases, participants were diagnosed by SARS-CoV-2-specific PCR. Study participants who acquired a SARS-CoV-2 infection during the study where excluded from the immunogenicity analyses of this manuscript.

For the control cohorts (mRNA; ChAd/mRNA) only individuals without prior SARS-CoV-2 exposure (self-reported) were included. Upon inclusion, participants were instructed to report clinical evidence of COVID-19-like symptoms or positive SARS-CoV-2 antigen and/or PCR test results. If participants acquired a SARS-CoV-2 infection, immunogenicity time points thereafter were excluded from the analyses of this manuscript.

### Blood sampling

A total of 452 peripheral blood samples were obtained from 76 donors. The blood sample collection schedule and the number of samples for each individual are shown in Supplementary Fig. 1. Blood was collected at T0 (baseline before vaccination), T1 (1–2 weeks), T2 (3-5 weeks), T3 (12 weeks), and T4 (17–29 weeks) post vaccination. Weeks refers to the time since the last vaccination. The exact time intervals between vaccinations and blood collections are shown in Supplementary Tables 2 and 3.

### PBMC and plasma isolation

Whole blood was collected in EDTA vacutainers. After centrifugation, plasma was removed and stored at −80 °C. PBMCs were isolated by density-gradient centrifugation using Ficoll-Histopaque (Sigma) or SepMate™ (Stemcell), cryopreserved, and stored in liquid nitrogen. Serum was collected using Gel monovettes with clotting activators, and stored at −20 °C. Additionally, whole blood was collected in lithium heparin monovettes and used for the IGRA assay within 10 h after collection.

### Bead-based multiplex immunoassay

A bead-based multiplex immunoassay was used to separately measure plasma antibody isotypes and IgG subclasses directed against the S1 and S2 subunits of the SARS-CoV-2 S-protein. For the detection of IgM, IgA and IgG isotypes, the MILLIPLEX® SARS-CoV-2 Antigen Panel 1 IgM/IgA/IgG kits (Merck KGaA) were used according to the manufacturer’s instructions with adjusted concentrations of detection antibodies. Briefly, magnetic beads coated with SARS-CoV-2 S1 and S2 antigens were added to a black, clear-bottom 96 well plate for each isotype. Plasma samples were added at a final dilution of 1:600 and plates were incubated on a plate shaker at 650 rpm at room temperature (RT) for 2 h. After washing, 45 µl of PE-anti-human IgG (#HC19-PEIGG), IgA (#HC19-PEIGA), or IgM (#HC19-PEIGM) conjugate was added to each well and incubated on a plate shaker at 650 rpm at RT for 1.5 h. After another washing step, the beads were resuspended in 150 µl of sheath fluid per well and stored overnight at 4°C. The plates were analyzed the next day using a Bio-Plex™ 200 system. For the detection of IgG subclasses, the MILLIPLEX® SARS-CoV-2 Antigen Panel 1 IgG kit (Merck KGaA) was used as described above, but detection antibodies were substituted with PE-conjugated antibodies specific to IgG1-4 (#SBA-9052-09, #SBA-9070-09, #SBA-9210-09, #SBA-9200-09; SouthernBiotech), added at a concentration of 0.65 µg/ml in 80 µl per well. For each isotype and subclass, wells without plasma samples were measured as controls for non-specific background signals and subtracted from the measured sample values. MFI values smaller less than 2 were set to 2. The results are shown as the mean of duplicate wells.

### SARS-CoV-2 VNT_100_

The serum neutralization capacity against SARS-CoV-2 was assessed by VNT_100_ (virus neutralization test) as described previously^53^. Briefly, vaccinee serum samples were heat-inactivated for 30 min at 56 °C and diluted in a two-fold dilution series (1:4–1:512) in 96-well cell culture plates, followed by addition of 100 plaque-forming units (PFU) of SARS-CoV-2 (German isolate BavPat1/2020; European Virus Archive Global #026 V-03883 (Genbank: MZ558051.1)). After 1 h of incubation at 37 °C, 2 × 10^4^ Vero C1008 cells (ATCC, Cat. no. CRL-1586, RRID: CVCL_0574) were added. Cytopathic effects were evaluated at day 4 post infection. Neutralization was defined as the absence of cytopathic effects, and the reciprocal neutralization titer was calculated from the highest serum dilution without cytopathic effects as a geometric mean based on three replicates. The lower limit of detection (LLOD) is a reciprocal titer of 8, corresponding to the first dilution of the respective serum.

### IgG ELISpot assay

SARS-CoV-2 S-specific B cells were analyzed using IgG ELISpot. To activate antibody secretion from B cells, PBMCs at a concentration of 2 × 10^6^/ml in R10 medium [RPMI 1640 (Sigma) supplemented with 10% fetal bovine serum (FBS) and 1% streptomycin/penicillin] containing 1% Hepes (Thermo Fisher Scientific), were stimulated with 0.5 µg/ml Resiquimod (R848, Mabtech) and 5 ng/ml interleukin-2 (IL-2, Mabtech) for 75 ± 1 h at 37 °C and 5% CO_2_. PVDF-MultiScreen-IP plates (Millipore) were treated with 35% ethanol and coated with anti-IgG capture antibody (15 µg/ml; #3850-2 A; Mabtech). After blocking with R10 containing 1% Hepes, pre-stimulated PBMCs were added to the wells at different concentrations and incubated for 16 h at 37 °C and 5% CO_2_. For the positive control (total IgG-secreting B cells), 1 × 10^4^ PBMCs were added per well, whereas numbers between 1 × 10^4^ and 8 × 10^5^ cells were used for the antigen-specific assay, depending on the time point post-vaccination. Biotinylated SARS-CoV-2 S-protein S1 or S2 subunit (S1: 0.1 µg/ml, S2: 0.2 µg/ml; SinoBiological) and anti-IgG detection antibody (1 µg/ml; #3850-2A; Mabtech) were added to detect antigen-specific and total IgG-secreting B cells, respectively. For spot development, streptavidin-ALP and BCIP/NBT-plus substrate solutions (Mabtech) were used according to the manufacturer’s protocol. The plates were analyzed using an AID EliSpot Reader System (AID GmbH). All samples were measured in duplicate and the mean was used for further analysis. Results below the LLOD (2 SFC/10^6^ PBMCs) were set to 1 SFC/10^6^ PBMCs.

### Peptide microarrays

To identify linear B cell epitopes in the S protein, we screened the sera of study participants using high-density peptide microarrays as described in^54^. The sequence of the SARS-CoV-2S protein (GenBank ID: MN908947.3) consisting of 1273 AA was mapped as a total of 634 overlapping 15-mer peptides with a lateral shift of two AA on peptide microarrays obtained from PEPperPRINT GmbH (Heidelberg, Germany). Serum samples were incubated on the arrays at 1:200 dilution, and IgG antibody interactions were then detected with fluorescently labeled secondary antibodies and quantified in arbitrary fluorescence units (AFU). Epitope binding was defined as positive if the mean AFU of three successive peptides was higher than 400 and 2.5-fold above the baseline before vaccination (if available).

### IFN-γ ELISpot assay

SARS-CoV-2 S-specific T cells were analyzed using the Human IFN-γ ELISpotPLUS (ALP) kit (Mabtech). After overnight resting in R10 containing 1% Hepes, PBMCs were seeded at 1.25 × 10^5^ cells/well in PVDF-MultiScreen-IP plates, pre-coated with anti-IFN-γ-mAB 1-D1K (#34206; Mabtech). Cells were then stimulated with a peptide pool (15-mers overlapping by 11 amino acids; Supplementary Data 2) spanning the SARS-CoV-2 S protein sequence (GenBank ID: MN908947.3) (2.5ug/ml in 0.1% dimethyl sulfoxide (DMSO); JPT Peptide Technologies) for 16 h at 37°C and 5% CO_2_. An equimolar concentration of DMSO was used as a negative control. Phytohemagglutinin (PHA) (1 μg/ml; Sigma) and CMV/EBV/Influenza (CEF) peptide pool (2 ug/ml; JPT Peptide Technologies) were used as positive control stimulations. Plates were then incubated with biotinylated anti-IFN-γ (1 μg/ml in PBS-0.5% FCS; clone mAb-7B6-1; #331010; Mabtech) for 2 h, followed by streptavidin-ALP (1:1000 in PBS-0.5% FCS; Mabtech) for 1 h at room temperature. Plates were developed using a substrate solution (BCIP/NBT; Mabtech). Spots were counted using an AID EliSpot Reader System (AID GmbH). Results are reported as SFC/10^6^ PBMCs, calculated by subtracting the mean count of triplicate negative control wells from the mean count of duplicate peptide-stimulated wells. Results were normalized to the total reactive T cells of each participant, using PHA stimulation as a positive control. Results below the LLOD (8 SFC/10^6^ PBMCs) were set to 4 SFC/10^6^ PBMCs.

### IFN-γ release T cell assay

IFN-γ secretion by S-specific T cells was analyzed in whole blood using a commercial, standardized IFN-γ release T cell assay (ET 2606-3003, Euroimmun, Lübeck, Germany). After a 20- to 24-h stimulation, IFN-γ was measured in the plasma using an IFN-γ ELISA (EQ 6841-9601, Euroimmun, Lübeck, Germany) according to the manufacturer’s instructions. IFN-γ secretion was quantified using a 5PL sigmoidal standard curve, and data are shown as background subtracted concentrations using an unstimulated control for each sample. Samples outside the standard curve were repeated at higher dilutions.

### Intracellular cytokine staining (ICS) assay

After overnight resting, PBMCs were stimulated with S peptides (2.5 μg/ml) for 7 h at 37 °C in the presence of Golgi-Plug, Golgi-Stop, and anti-CD28/CD49 (1:100; #9035982; BD Biosciences) in 96-well V-bottom plates (Sarstedt). For each sample, cells incubated with an equimolar amount of DMSO (0.1%) and Phorbol-12-myristate-13-acetate (50 ng/ml) plus ionomycin (0.5 μg/ml) served as negative and positive controls, respectively. Cells were then washed and stained with an antibody mix of anti-CD3-BUV395 (1:100; #564001; BD Biosciences), anti-CD4-AF700 (1:50; #300526; BioLegend), anti-CD19-BV510 (1:100; #302242; BioLegend), anti-CD14-BV510 (1:100; #301842; BioLegend), anti-CD8-APC-Cy7 (1:100; #344714; BioLegend), anti-CCR7-AF647 (1:50; #353218; BioLegend), anti-CD45RO-FITC (1:33; #304242; BioLegend), and Zombie Aqua™ Fixable Viability Kit (1:500; #423101; BioLegend) in FACS buffer [PBS supplemented with 2% FBS and 2 mM EDTA] for 15 min at 37 °C. Subsequently, cells were fixed (eBioscience™), washed, and stained with intracellular markers anti-IFN-γ-PE-Cy7 (1:50; #506518; BioLegend), anti-TNF-α-PE/Dazzle™ 594 (1:100; #50296; BioLegend) and IL-2-PerCP-Cy5.5 (1:25; #500322, BioLegend) in PERM buffer (eBioscience™) at RT for 15 min. Samples were stored in FACS buffer at 4 °C and analyzed on the BD Fortessa the following day. A representative gating strategy is shown in Supplementary Fig. 4. Cytokine-secreting memory T cells were identified by excluding CCR7^+^/CD45RO^-^ naïve T cells and then gating the individual cytokines on CD4^+^ and CD8^+^ T cells separately. Results are shown as background (DMSO) subtracted data (Fig. 6). Multifunctional profiles were identified by Boolean gating. Results of the Boolean gates were summed for each sample, based on the number of functions.

### Statistical analysis

Statistical testing was performed based on R (v4.2.0), using nonparametric tests due to the small sample sizes per cohort. Two-tailed Wilcoxon matched-pairs signed rank test and Mann-Whitney *U* test were used for testing paired and unpaired samples, respectively, as indicated in Supplementary Tables 9–12. The calculated p-values were adjusted for multiple comparisons within each figure using the Benjamini & Hochberg correction. Due to the small numbers of study participants per cohort, and the different sample sizes per group, p-values have to be interpreted with caution. Correlations were calculated with GraphPad Prism software (v9.5.1) using non-parametric Spearman’s correlation. The significance level for all statistical tests was set to 0.05. Analysis of flow cytometry data was done using FlowJo (v.10.8.1). Figures were created using GraphPad Prism (v9.5.1), R (v4.2.0), and BioRender.com. The sample size for each analysis is listed in Supplementary Tables 4–8.

### Reporting summary

Further information on research design is available in the Nature Research Reporting Summary linked to this article.

### Supplementary information

Supplementary Information

Supplementary Data 1

Supplementary Data 2

Reporting summary

## Data Availability

The datasets used and/or analyzed during the current study are available from the corresponding author upon reasonable request.
