# Supplementary material for: MVA-based vaccine candidates encoding the native or prefusion-stabilized SARS-CoV-2 spike reveal differential immunogenicity in humans
Source: NPJ Vaccines. 2024 Jan 26;9:20. doi: 10.1038/s41541-023-00801-z (PMC10817990; doi:10.1038/s41541-023-00801-z)
Supplement: Supplementary file 1 — Supplementary Information [file 41541_2023_801_MOESM1_ESM.pdf]

Supplementary Information

Supplementary Note 1 | Background and timeline of (pre-)clinical development of rMVA-based vaccines.

Upon the emergence of SARS-CoV-2 at the end of 2019 and its subsequent global spread in 2020, the MVA-SARS-2-S (MVA-S) vaccine candidate was constructed. The safety and efficacy of MVA-S were tested in a pre-clinical model, where BALB/c mice of two dose groups were vaccinated in a prime-boost regimen. Following booster immunization, S-binding serum IgG and neutralizing titers were induced in all animals. Additionally, robust, Th1-skewed, S-specific T cell responses were measured in both, the low and the high dose groups. Upon SARS-CoV-2 challenge, all animals were protected from lung damage in the absence of detectable infectious viruses in the lungs. These data illustrated preclinical safety and efficacy and provided evidence that MVA-S is a promising vaccine candidate for evaluation in humans [1].

MVA-S then entered phase 1a of clinical evaluation (ClinicalTrials.gov: NCT04569383) in October 2020 to test the safety and immunogenicity of two ascending doses in healthy adults (MVA-S/mRNA cohort described in this manuscript). However, an interim analysis revealed that S-binding antibody titers were lower than expected, and only 33% of individuals reached seroconversion [2]. The clinical study was then amended such that participants received two doses of the (by then licensed) BNT162b2 mRNA vaccine at least six months after completion of the primary vaccination series with MVA-S. Safety and immunogenicity monitoring were continued.

Subsequently, an optimized vaccine candidate expressing the prefusion-stabilized S-protein, namely MVA-SARS-2-ST (MVA-ST) was constructed. MVA-ST was then tested in preclinical models for direct comparison with the MVA-S candidate. MVA-ST induced higher S-binding, RBD-specific, and neutralizing titers than MVA-S in mice. Analysis of antibody responses against the S1 and S2 subunits showed that MVA-S and MVA-ST induced comparable titers of S2-specific IgG. However, MVA-ST induced significantly higher S1-specific titers. Upon challenge, Syrian hamsters vaccinated with MVA-S or MVA-ST both showed no signs of clinical disease, but reduction of viral load was more pronounced in the MVA-ST-vaccinated group [2].

Thus, MVA-ST entered phase 1b clinical evaluation (ClinicalTrials.gov: NCT04895449) in June 2021 to test the safety and immunogenicity of two ascending doses in healthy adults (MVA-ST cohort described in this manuscript). Due to the progression of the pandemic and the availability of licensed vaccines at that time, an additional group of previously SARS-CoV-2-vaccinated individuals (the mRNA/MVA-ST cohort described in this manuscript) was included in the trial, to test MVA-ST as a booster vaccination.

Because of this timeline, clinical studies were performed for both MVA-S and MVA-ST, providing the unique opportunity to directly compare the immunogenicity in humans of two vaccine candidates based on the same viral vector but different S-protein conformations (as described in this manuscript).

1. Tscherne, A. *et al.* Immunogenicity and efficacy of the COVID-19 candidate vector vaccine MVA-SARS-2-S in preclinical vaccination. *Proc. Natl. Acad. Sci. U. S. A.* **118**, e2026207118; doi: 10.1073/pnas.2026207118 (2021).

2. Meyer zu Natrup, C. *et al.* Stabilized recombinant SARS-CoV-2 spike antigen enhances vaccine immunogenicity and protective capacity. *J. Clin. Invest.* ***132***, e159895; doi: 10.1172/JCI159895 (2022).

Supplementary Note 2 | MVA-SARS-2 Study Group

Amelie Alberti, Marie-Louise Dieck, Stefanie Gräfe, Cordula Grüttner, Jana Kochmann, Niclas Renevier, Monika Rottstegge, Maren Sandkuhl, Claudia Schlesner, Yashin Simsek, Paulina Tarnow. All members of the MVA-SARS-2 Study Group are affiliated with the Institute for Infection Research and Vaccine Development (IIRVD) at the University Medical Center Hamburg-Eppendorf, the Department for Clinical Immunology of Infectious Diseases at the Bernhard Nocht Institute for Tropical Medicine (BNITM), and the German Centre for Infection Research (DZIF) partner site Hamburg-Lübeck-Borstel-Riems.

Supplementary Figures

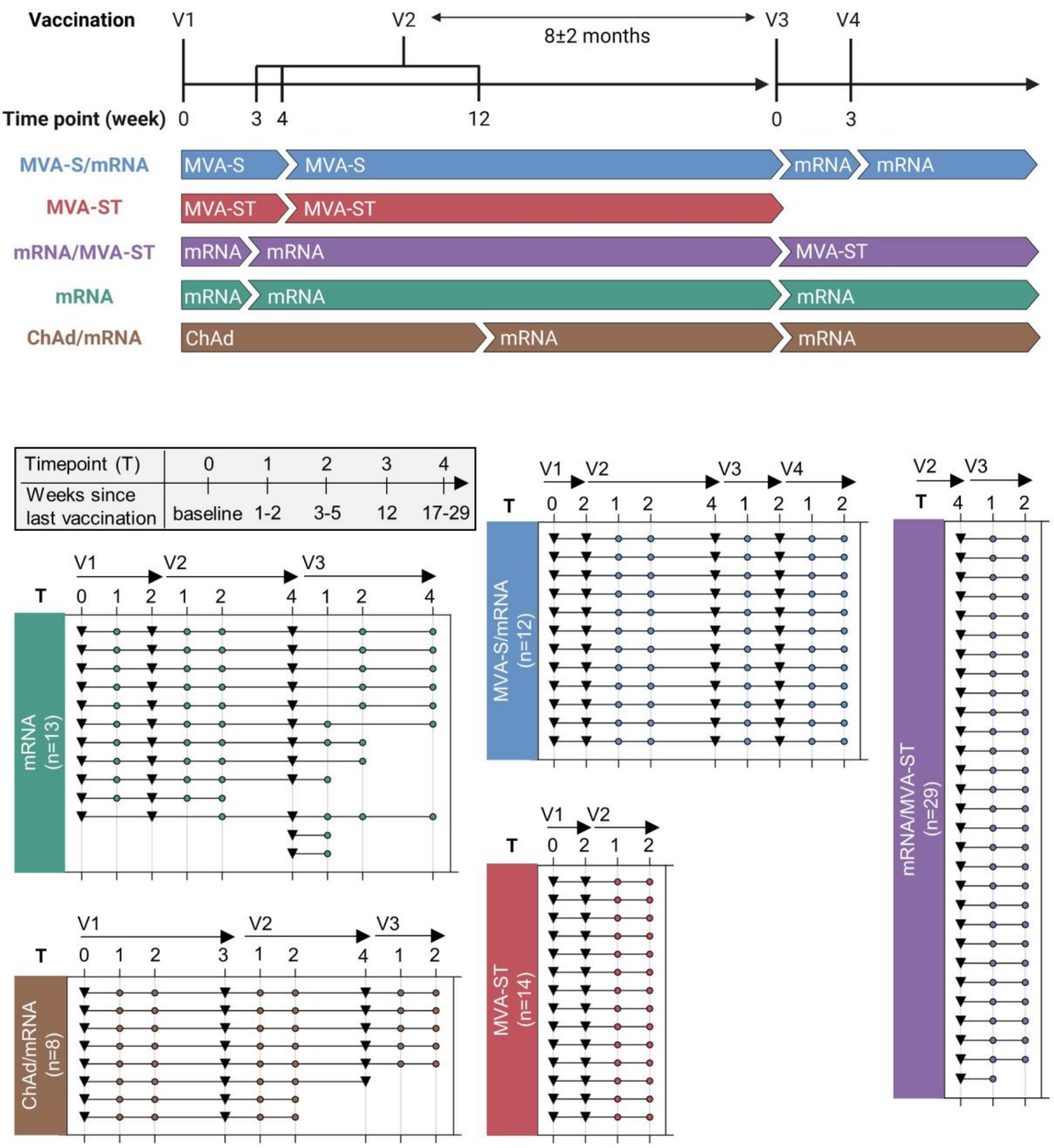

Supplementary Figure 1 | Longitudinal blood sampling. Participants of five study cohorts received up to 4 vaccinations (V1 to V4) with different COVID-19 vaccines. Time intervals between vaccinations differed between the cohorts and are indicated in the upper panel. The vaccines administered in this study include the two rMVA-based vaccine candidates MVA-SARS-2-S (MVA-S) and MVA-SARS-2-ST (MVA-ST), as well as the licensed vaccines BNT162b2 and mRNA-1273 (together referred to as mRNA) and ChAdOx1 nCov-19 (ChAd). Blood samples were collected at different time points after vaccination, labeled as T0 (baseline), T1 (1-2 weeks), T2 (3-5 weeks), T3 (12 weeks), and T4 (17-29 weeks), referring to the time since last vaccination (V1-V4). Time points of longitudinal blood sampling are shown in the lower panel for each participant of the different cohorts as colored dots. Vaccinations are shown as black triangles.

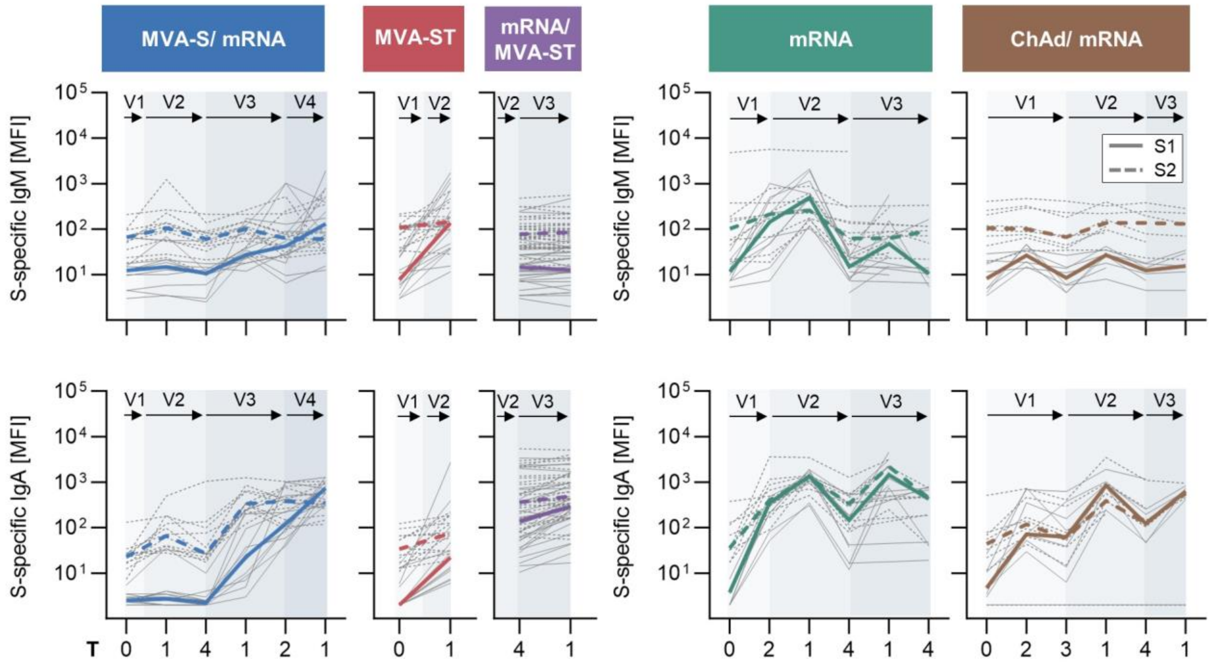

Supplementary Figure 2 | S1/S2-specific IgM and IgA response. a S1- and S2- specific IgM (top) and IgA (bottom) responses of the different study cohorts measured at baseline and longitudinally after each vaccination. Colored lines depict median MFI (measured by bead-based multiplex immunoassay). Grey lines show dynamics of each study participant.

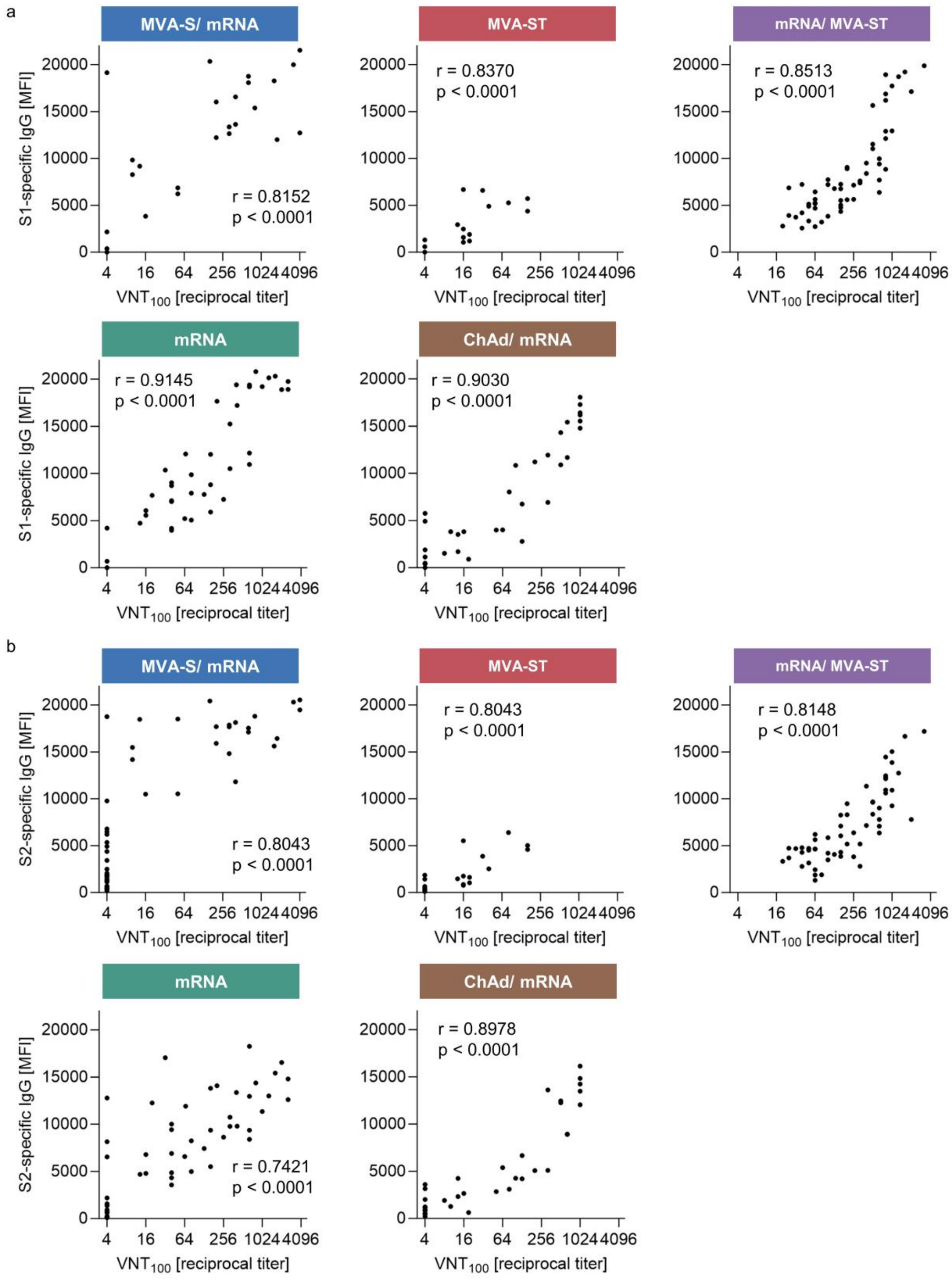

Supplementary Figure 3 | Neutralization capacity versus IgG antibody response. Spearman correlation of serum neutralizing capacity (measured by SARS-CoV-2 virus neutralization test, VNT_{100}) with S1- (a) and S2- (b) specific IgG stratified by study cohort.

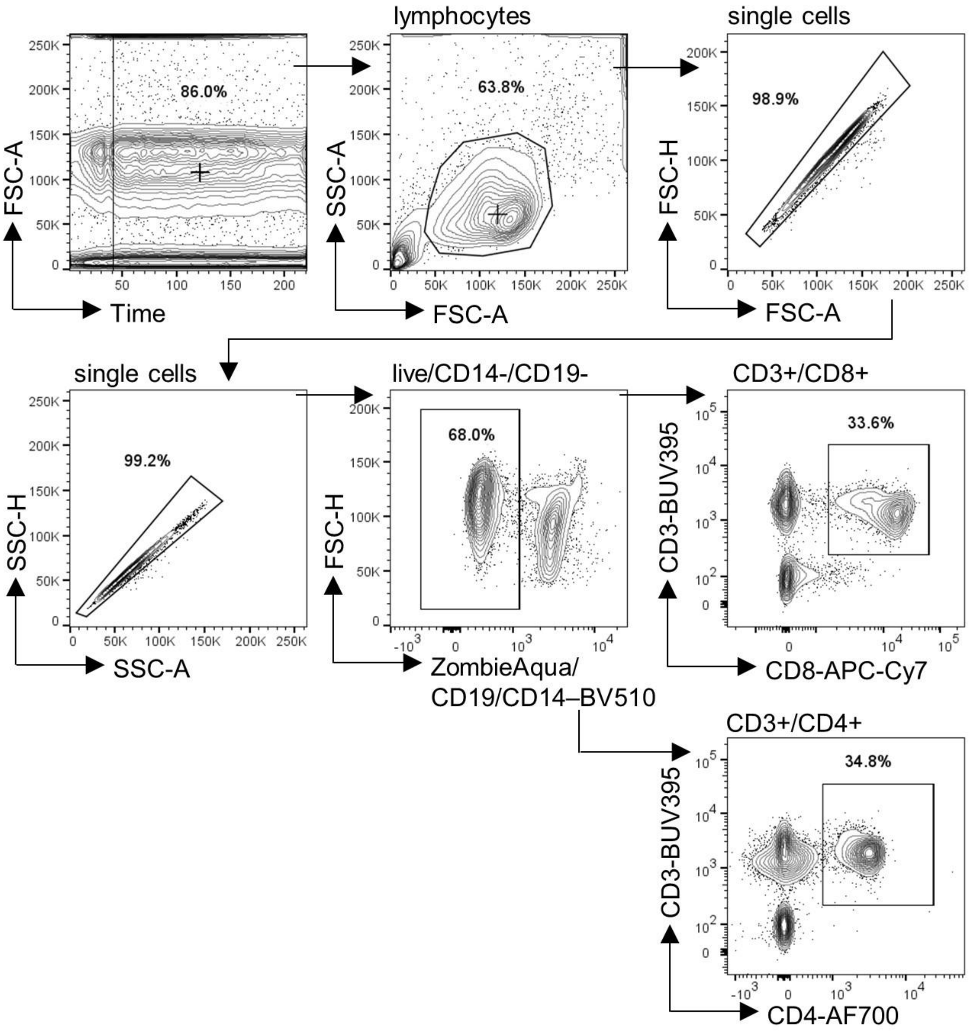

Supplementary Figure 4 | T cell gating strategy. Gating strategy for intracellular cytokine staining T cell assay (related to Fig. 6). Contour plots show representative data from an individual of the ChAd/mRNA cohort at V2:T2.

Supplementary Tables

Supplementary Table 1 | Baseline characteristics of study participants

	mRNA n=13	ChAd/mRNA n=8	MVA-S/mRNA n=12	MVA-ST n=14	mRNA/MVA-ST n=29
**Sex**					
Female	8 (62%)	7 (88%)	4 (33%)	9 (64%)	18 (62%)
Male	5 (38%)	1 (12%)	8 (67%)	5 (36%)	11 (38%)
**Age**					
mean, years	33.2 (8.9)	32.3 (5.9)	37.8 (9·0)	41 (11.1)	32.0 (11.2)
range, years	23-51	24 - 44	21 - 51	22 - 62	19 - 64
**BMI**					
kg/m^2	21.4 (2.4)	21.7 (1.3)	24.9 (3.4)	24.0 (3.45)	24.6 (2.9)
Data is shown in mean (SD), unless otherwise indicated. BMI=body-mass index. BMI of n=2 of the mRNA cohort missing.					

Supplementary Table 2 | Time interval between vaccinations

	mRNA n=13	ChAd/mRNA n=8	MVA-S/mRNA n=12	MVA-ST n=14	mRNA/MVA-ST n=29
**Vaccination interval**					
V1-V2	21 (21-40)	84 (77-84)	28	28	
V2-V3	252 (196-291)	183 (170-211)	203 (185-211)		220.5 (187-364)
V3-V4			22 (21-28)		
Data is shown as median days (range)					

Supplementary Table 3 | Blood collection time since last vaccination

	mRNA n=13	ChAd/mRNA n=8	MVA-S/mRNA n=12	MVA-ST n=14	mRNA/MVA-ST n=29
**V1**					
T1	7	7 (7-8)	-	-	-
T2	21 (20-23)	28 (28-31)	28 (28-29)	28	-
T3	-	80.5 (56-83)	-	-	-
**V2**					
T1	7 (7-8)	7 (7-10)	14 (12-16)	14 (14-16)	-
T2	35 (34-38)	36.5 (29-42)	29 (27-33)	28 (28-35)	-
T4	164 (147-169)	169 (168-171)	203 (185-211)	-	220.5 (187-364)
**V3**					
T1	8.5 (7-15)	7 (7-8)	7	-	7 (7-14)
T2	28 (28-36)	28 (27-32)	21.5 (21-28)		28 (25-34)
T4	124.5 (119-137)	-	-	-	-
**V4**					
T1	-	-	7 (6-7)	-	-
T2	-	-	28 (28-33)		-
Data is shown as median days (range)					

Supplementary Table 4 | Number of samples – mRNA cohort

Assay	Timepoint	V1:T0	V1:T1	V1:T2	V2:T1	V2:T2	V2:T4	V3:T1	V3:T2	V3:T4
B cell	Antibody isotypes and subclasses	10		10	10		13	6		7
	IgG ELISpot	10	10	10	10		13	5		7
	IgG epitope array	9			9					
T cell	IFNγ ELISpot	10	10	10	10	10	13	6	7	7
	Intracellular cytokine staining	10		10		10				

Supplementary Table 5 | Number of samples – ChAd/mRNA cohort

Assay	Timepoint	V1:T0	V1:T1	V1:T2	V1:T3	V2:T1	V2:T2	V2:T4	V3:T1	V3:T2
B cell	Antibody isotypes and subclasses	8		8	8	8		6	5	
	IgG ELISpot	8	8	8	8	8		6	5	
	IgG epitope array	5				5			5	
T cell	IFNγ ELISpot	8	8	8	8	8	8	6	5	5
	Intracellular cytokine staining	8		8			8			

Supplementary Table 6 | Number of samples – MVA-S/mRNA cohort

Assay	Timepoint	V1:T0	V1:T2	V2:T1	V2:T2	V2:T4	V3:T1	V3:T2	V4:T1	V4:T2
B cell	Antibody isotypes and subclasses	12		12		12	12	12	12	
	S1-specific IgG ELISpot	12		12		12	S1: 12, S2: 11	S1: 12, S2: 11	12	
	Epitope array	5		5					5	
T cell	IFNγ ELISpot	12	10	11	12	12	12	12	12	12
	Intracellular cytokine staining	12						12		12

Supplementary Table 7 | Number of samples – MVA-ST

Assay	Timepoint	V1:T0	V1:T2	V2:T1	V2:T2
B cell	Antibody isotypes and subclasses	14		14	
	IgG ELISpot	S1: 14, S2: 13		14	
	IgG epitope array	7		7	
T cell	IFNγ ELISpot	14	14	14	14
	Intracellular cytokine staining				

Supplementary Table 8 | Number of samples – mRNA/MVA-ST

Assay	Timepoint	V2:T4	V3:T1	V3:T2
B cell	Antibody isotypes and subclasses	29	29	
	IgG ELISpot	28	29	
	IgG epitope array			
T cell	IFNγ ELISpot	29	29	28
	Intracellular cytokine staining			

Supplementary Table 9 | Statistical analysis of S1/S2-specific IgG responses, related to Figure 2.

Figure/ panel	parameter	cohort	time point	test	adjusted p-value	p-value summary
2a	S1	MVA-S/mRNA	V2T1 vs T0	Wilcoxon	0.0104	*
2a	S1	MVA-S/mRNA	V2T4 vs T0	Wilcoxon	0.5907	ns
2a	S1	MVA-ST	V2T1 vs T0	Wilcoxon	0.0005	***
2a	S1	mRNA	V2T1 vs T0	Wilcoxon	0.0036	**
2a	S1	ChAd/mRNA	V2T1 vs T0	Wilcoxon	0.0119	*
2a	S1	mRNA/MVA-ST	V3T1 vs V2T4	Wilcoxon	0.0007	***
2a	S1	mRNA	V3T1 vs V2T4	Wilcoxon	0.0421	*
2a	S1	ChAd/mRNA	V3T1 vs V2T4	Wilcoxon	0.0754	ns
2a	S2	MVA-S/mRNA	V2T1 vs T0	Wilcoxon	0.0012	**
2a	S2	MVA-S/mRNA	V2T4 vs T0	Wilcoxon	0.0030	**
2a	S2	MVA-ST	V2T1 vs T0	Wilcoxon	0.0005	***
2a	S2	mRNA	V2T1 vs T0	Wilcoxon	0.0036	**
2a	S2	ChAd/mRNA	V2T1 vs T0	Wilcoxon	0.0119	*
2a	S2	mRNA/MVA-ST	V3T1 vs V2T4	Wilcoxon	0.0018	**
2a, 2d	S2	mRNA	V3T1 vs V2T4	Wilcoxon	0.0421	*
2a	S2	ChAd/mRNA	V3T1 vs V2T4	Wilcoxon	0.0754	ns
2b	S1	MVA-S/mRNA vs MVA-ST	V2T1	Mann-Whitney	<0.0001	****
2b	S1	MVA-S/mRNA vs mRNA	V2T1	Mann-Whitney	<0.0001	****
2b	S1	MVA-S/mRNA vs ChAd/mRNA	V2T1	Mann-Whitney	<0.0001	****
2b	S1	MVA-ST vs mRNA	V2T1	Mann-Whitney	<0.0001	****
2b	S1	MVA-ST vs ChAd/mRNA	V2T1	Mann-Whitney	0.0006	***
2b	S1	mRNA vs ChAd/mRNA	V2T1	Mann-Whitney	0.0104	*
2b	S2	MVA-S/mRNA vs MVA-ST	V2T1	Mann-Whitney	0.5952	ns
2b	S2	MVA-S/mRNA vs mRNA	V2T1	Mann-Whitney	<0.0001	****
2b	S2	MVA-S/mRNA vs ChAd/mRNA	V2T1	Mann-Whitney	0.0008	***
2b	S2	MVA-ST vs mRNA	V2T1	Mann-Whitney	<0.0001	****
2b	S2	MVA-ST vs ChAd/mRNA	V2T1	Mann-Whitney	0.0005	***
2b	S2	mRNA vs ChAd/mRNA	V2T1	Mann-Whitney	0.3000	ns
2c	S1	MVA-S/mRNA vs mRNA	V3T2 vs V1T2	Mann-Whitney	0.0807	ns
2c	S2	MVA-S/mRNA vs mRNA	V3T2 vs V1T2	Mann-Whitney	0.0014	**
2d	S1	mRNA/MVA-ST, LD	V3T1 vs V2T4	Wilcoxon	0.0551	ns

2d	S1	mRNA/MVA-ST, MD	V3T1 vs V2T4	Wilcoxon	0.0285	*
2d	S1	mRNA/MVA-ST, HD	V3T1 vs V2T4	Wilcoxon	0.0882	ns
2e	S1	mRNA/MVA-ST, low baseline	V3T1 vs V2T4	Wilcoxon	0.0006	***
2e	S1	mRNA/MVA-ST, high baseline	V3T1 vs V2T4	Wilcoxon	0.3792	ns

Supplementary Table 10 | Statistical analysis of S1/S2-specific B cell responses, related to Figure 4.

Figure/ panel	parameter	cohort	time point	test	adjusted p-value	p-value summary
4a	S1	MVA-S/mRNA	V2T1 vs T0	Wilcoxon	0.0298	*
4a	S1	MVA-ST	V2T1 vs T0	Wilcoxon	0.0156	*
4a	S1	mRNA	V1T2 vs T0	Wilcoxon	0.0201	*
4a	S1	mRNA	V2T1 vs T0	Wilcoxon	0.0066	**
4a	S1	ChAd/mRNA	V1T2 vs T0	Wilcoxon	0.0170	*
4a	S1	ChAd/mRNA	V2T1 vs T0	Wilcoxon	0.0170	*
4a	S1	mRNA/MVA-ST	V3T1 vs V2T4	Wilcoxon	0.8615	ns
4a	S1	mRNA	V3T1 vs V2T4	Wilcoxon	0.0925	ns
4a	S1	ChAd/mRNA	V3T1 vs V2T4	Wilcoxon	0.0925	ns
4a	S2	MVA-S/mRNA	V2T1 vs T0	Wilcoxon	0.0018	**
4a	S2	MVA-ST	V2T1 vs T0	Wilcoxon	0.0078	**
4a	S2	mRNA	V1T2 vs T0	Wilcoxon	0.0419	*
4a	S2	mRNA	V2T1 vs T0	Wilcoxon	0.0156	*
4a	S2	ChAd/mRNA	V1T2 vs T0	Wilcoxon	0.0419	*
4a	S2	ChAd/mRNA	V2T1 vs T0	Wilcoxon	0.0170	*
4a	S2	mRNA/MVA-ST	V3T1 vs V2T4	Wilcoxon	0.3383	ns
4a	S2	mRNA	V3T1 vs V2T4	Wilcoxon	0.2417	ns
4a	S2	ChAd/mRNA	V3T1 vs V2T4	Wilcoxon	0.0925	ns
4b	S1	MVA-S/mRNA vs MVA-ST	V2T1	Mann-Whitney	0.2417	ns
4b	S1	MVA-S/mRNA vs mRNA	V2T1	Mann-Whitney	0.0001	***
4b	S1	MVA-S/mRNA vs ChAd/mRNA	V2T1	Mann-Whitney	0.0003	***
4b	S1	MVA-ST vs mRNA	V2T1	Mann-Whitney	0.0004	***
4b	S1	MVA-ST vs ChAd/mRNA	V2T1	Mann-Whitney	0.0008	***
4b	S1	mRNA vs ChAd/mRNA	V2T1	Mann-Whitney	0.8290	ns
4b	S2	MVA-S/mRNA vs MVA-ST	V2T1	Mann-Whitney	0.0228	*
4b	S2	MVA-S/mRNA vs mRNA	V2T1	Mann-Whitney	0.0017	**
4b	S2	MVA-S/mRNA vs ChAd/mRNA	V2T1	Mann-Whitney	0.0005	***
4b	S2	MVA-ST vs mRNA	V2T1	Mann-Whitney	0.0004	***
4b	S2	MVA-ST vs ChAd/mRNA	V2T1	Mann-Whitney	0.0008	***
4b	S2	mRNA vs ChAd/mRNA	V2T1	Mann-Whitney	0.0973	ns
4c	S1	MVA-S/mRNA vs mRNA	V3T2 vs V1T2	Mann-Whitney	1.0000	ns
4c	S2	MVA-S/mRNA vs mRNA	V3T2 vs V1T2	Mann-Whitney	0.0008	***
4d	S1	mRNA/MVA-ST_LD	V3T1 vs V2T4	Wilcoxon	0.8362	ns
4d	S1	mRNA/MVA-ST_MD	V3T1 vs V2T4	Wilcoxon	0.3653	ns
4d	S1	mRNA/MVA-ST_HD	V3T1 vs V2T4	Wilcoxon	0.1448	ns
4e	S1	mRNA/MVA-ST_low baseline	V3T1 vs V2T4	Wilcoxon	0.3515	ns
4e	S1	mRNA/MVA-ST_high baseline	V3T1 vs V2T4	Wilcoxon	0.3653	ns

Supplementary Table 11 | Statistical analysis of T cell responses as measured by ELISpot, related to Figure 5.

Figure/ panel	cohort	time point	test	adjusted p-value	p-value summary
5a	MVA-S/mRNA	V2T1 vs T0	Wilcoxon	0.1782	ns
5a	MVA-ST	V2T1 vs T0	Wilcoxon	0.0249	*
5a	mRNA	V1T2 vs T0	Wilcoxon	0.0714	ns
5a	ChAd/mRNA	V1T2 vs T0	Wilcoxon	0.0165	*
5a	mRNA/MVA-ST	V3T2 vs V2T4	Wilcoxon	0.0012	**
5a, 5g	mRNA	V3T2 vs V2T4	Wilcoxon	0.0990	ns
5a	ChAd/mRNA	V3T2 vs V2T4	Wilcoxon	0.0848	ns
5d	MVA-S/mRNA vs MVA-ST	V2T1	Mann-Whitney	0.0021	**
5d	MVA-S/mRNA vs mRNA	V2T1	Mann-Whitney	0.0012	**
5d	MVA-S/mRNA vs ChAd/mRNA	V2T1	Mann-Whitney	0.0012	**
5d	MVA-ST vs mRNA	V2T1	Mann-Whitney	0.0021	**
5d	MVA-ST vs ChAd/mRNA	V2T1	Mann-Whitney	0.0023	**
5d	mRNA vs ChAd/mRNA	V2T1	Mann-Whitney	0.9654	ns
5f	MVA-S/mRNA vs mRNA	V3T1 vs V1T1	Mann-Whitney	0.0211	*
5g	mRNA/MVA-ST_LD	V3T2 vs V2T4	Wilcoxon	0.4127	ns
5g	mRNA/MVA-ST_MD	V3T2 vs V2T4	Wilcoxon	0.0093	**
5g	mRNA/MVA-ST_HD	V3T2 vs V2T4	Wilcoxon	0.0714	ns
5h	mRNA/MVA-ST_low baseline	V3T2 vs V2T4	Wilcoxon	0.0012	**
5h	mRNA/MVA-ST_high baseline	V3T2 vs V2T4	Wilcoxon	0.4127	ns

Supplementary Table 12 | Statistical analysis of T cell responses as measured by ICS, related to Figure 6.

Figure/ panel	parameter	cohort	time point	test	adjusted p-value	p-value summary
6d	CD4_IFNg	MVA-S/mRNA	V4T2 vs V1D0	Wilcoxon	0.0059	**
6d	CD4_IFNg	mRNA	V2T2 vs V1D0	Wilcoxon	0.0275	*
6d	CD4_IL2	MVA-S/mRNA	V4T2 vs V1D0	Wilcoxon	0.0275	*
6d	CD4_IL2	mRNA	V2T2 vs V1D0	Wilcoxon	0.0117	*
6d	CD4_TNFa	MVA-S/mRNA	V4T2 vs V1D0	Wilcoxon	0.0346	*
6d	CD4_TNFa	mRNA	V2T2 vs V1D0	Wilcoxon	0.0418	*
6d	CD8_IFNg	MVA-S/mRNA	V4T2 vs V1D0	Wilcoxon	0.0441	*
6d	CD8_IFNg	mRNA	V2T2 vs V1D0	Wilcoxon	0.0418	*
6d	CD4_IL2	MVA-S/mRNA	V4T2 vs V1D0	Wilcoxon	0.6726	ns
6d	CD4_IL2	mRNA	V2T2 vs V1D0	Wilcoxon	0.1122	ns
6d	CD8_TNFa	MVA-S/mRNA	V4T2 vs V1D0	Wilcoxon	0.2020	ns
6d	CD8_TNFa	mRNA	V2T2 vs V1D0	Wilcoxon	0.0774	ns
