# Supplementary material for: MVA-based vaccine candidates encoding the native or prefusion-stabilized SARS-CoV-2 spike reveal differential immunogenicity in humans
Source: NPJ Vaccines. 2024 Jan 26;9:20. doi: 10.1038/s41541-023-00801-z (PMC10817990; doi:10.1038/s41541-023-00801-z)
Supplement: Supplementary file 4 — Reporting summary [file 41541_2023_801_MOESM4_ESM.pdf]

nature portfolio

Corresponding author(s): Marylyn M. Addo, Leonie Mayer

Last updated by author(s): Nov 8, 2023

Reporting Summary

Nature Portfolio wishes to improve the reproducibility of the work that we publish. This form provides structure for consistency and transparency in reporting. For further information on Nature Portfolio policies, see our Editorial Policies and the Editorial Policy Checklist.

Statistics

For all statistical analyses, confirm that the following items are present in the figure legend, table legend, main text, or Methods section.

n/a	Confirmed	
☐	☒	The exact sample size (n) for each experimental group/condition, given as a discrete number and unit of measurement
☐	☒	A statement on whether measurements were taken from distinct samples or whether the same sample was measured repeatedly
☐	☒	The statistical test(s) used AND whether they are one- or two-sided<br>*Only common tests should be described solely by name; describe more complex techniques in the Methods section.*
☒	☐	A description of all covariates tested
☐	☒	A description of any assumptions or corrections, such as tests of normality and adjustment for multiple comparisons
☐	☒	A full description of the statistical parameters including central tendency (e.g. means) or other basic estimates (e.g. regression coefficient) AND variation (e.g. standard deviation) or associated estimates of uncertainty (e.g. confidence intervals)
☐	☒	For null hypothesis testing, the test statistic (e.g. F, t, r) with confidence intervals, effect sizes, degrees of freedom and P value noted<br>*Give P values as exact values whenever suitable.*
☒	☐	For Bayesian analysis, information on the choice of priors and Markov chain Monte Carlo settings
☒	☐	For hierarchical and complex designs, identification of the appropriate level for tests and full reporting of outcomes
☒	☐	Estimates of effect sizes (e.g. Cohen's d, Pearson's r), indicating how they were calculated

Our web collection on statistics for biologists contains articles on many of the points above.

Software and code

Policy information about availability of computer code

Data collection	Bio-Plex Manager™ Software v6.2 (build 175, Bio-Rad Laboratories, Inv.), Elispot Reader v7.0 (build 16577, AID GmbH), FACSDiva Software v8.0.1 (BD Biosciences)
Data analysis	FlowJo v10.8.1 (FlowJo, LLC), GraphPad Prism v9.5.1 (build 733, Dotmatics), R v. 4.2.0 (The R Foundation), RStudio 2023.06.1 (Posit PBC) Excel v1808 (build 10403.20013, Microsoft)

For manuscripts utilizing custom algorithms or software that are central to the research but not yet described in published literature, software must be made available to editors and reviewers. We strongly encourage code deposition in a community repository (e.g. GitHub). See the Nature Portfolio guidelines for submitting code & software for further information.

Data

Policy information about availability of data

All manuscripts must include a data availability statement. This statement should provide the following information, where applicable:

- Accession codes, unique identifiers, or web links for publicly available datasets
- A description of any restrictions on data availability
- For clinical datasets or third party data, please ensure that the statement adheres to our policy

The datasets used and/or analysed during the current study are available from the corresponding author on reasonable request.

Research involving human participants, their data, or biological material

Policy information about studies with human participants or human data. See also policy information about sex, gender (identity/presentation), and sexual orientation and race, ethnicity and racism.

Reporting on sex and gender	Biological sex was self-reported by study participants (see supplementary material), but data was not stratified by sex in our study due to cohort size. Efforts were made to include equal numbers of men and women as prespecified by the study protocols.
Reporting on race, ethnicity, or other socially relevant groupings	We did not report on race or ethnicity in our study.
Population characteristics	Only healthy adults (aged 18-65) were included into our study. Inclusion and exclusion criteria are reported in the study protocols of the clinical trials and the methods section of our manuscript.
Recruitment	For the MVA-S and MVA-ST clinical trials, participants were recruited via public advertisement. For the control cohorts, participants were recruited from an established internal institutional cohort of the University Medical Centre Hamburg-Eppendorf and the Bernhard Nocht Institute for Tropical Medicine, Hamburg, Germany.
Ethics oversight	The MVA phase 1 clinical trials were reviewed and approved by the National Competent Authority (Paul-Ehrlich-Institute, EudraCT numbers 2020-003875-16; 2021-000548-23) and the Ethics Committee of the Hamburg Medical Association (reference numbers 2020-10164-AMG-ff; 2021-100621-AMG-ff), conducted under the sponsorship of the University Medical Center Hamburg-Eppendorf (Hamburg, Germany) in accordance with ICH GCP and the EU directives 2001/20/EC and 2001/83/EC, and are registered at ClinicalTrials.gov. (NCT04569383; NCT04895449). The Ethics Committee of the Hamburg Medical Association approved the clinical study with licensed vaccines (reference number: 2020-10376-BO-ff).

Note that full information on the approval of the study protocol must also be provided in the manuscript.

Field-specific reporting

Please select the one below that is the best fit for your research. If you are not sure, read the appropriate sections before making your selection.

☒ Life sciences ☐ Behavioural & social sciences ☐ Ecological, evolutionary & environmental sciences

For a reference copy of the document with all sections, see nature.com/documents/nr-reporting-summary-flat.pdf

Life sciences study design

All studies must disclose on these points even when the disclosure is negative.

Sample size	Part of this manuscript are three cohorts receiving MVA-based COVID-19 vaccines as part of clinical trials with 12, 14 and 29 study participants, respectively. In addition, we report data from two observational control cohorts receiving licensed vaccine regimens (n = 13; n = 8). Small sample sizes are inherent to early phase clinical trials intended as proof-of-concept studies. In this specific case, the inclusion of additional partipants was hindered by the rapid progression of the COVID-19 pandemic and vaccination campaigns. The strength of this study is the longitudinal sampling of each study participant, and the draw-backs of the limited sample size are addressed in the statistics section.
Data exclusions	Data included in the paper had to conform with pre-defined quality control standards. Measurements that did not conform with these criteria were excluded or repeated. Sample sizes for each assay are indicated in the supplement.
Replication	All assays were performed according to established protocols. Number of replicates are defined in the figure legends and/or methods section.
Randomization	In the MVA phase 1 trials, participants were successively assigned to treatment arms according to the clinical trial protocols. In the control cohorts, participants received licensed vaccine regimens according to the health authorities' recommendations. As this was an observational study, randomization was not applicable.
Blinding	Since the studies on MVA vaccines represented open-label phase 1 trials, no blinding was performed according to the clinical trial protocols.

Reporting for specific materials, systems and methods

We require information from authors about some types of materials, experimental systems and methods used in many studies. Here, indicate whether each material, system or method listed is relevant to your study. If you are not sure if a list item applies to your research, read the appropriate section before selecting a response.

Materials & experimental systems

n/a	Involved in the study
☐	☒ Antibodies
☐	☒ Eukaryotic cell lines
☒	☐ Palaeontology and archaeology
☒	☐ Animals and other organisms
☐	☒ Clinical data
☒	☐ Dual use research of concern
☒	☐ Plants

Methods

n/a	Involved in the study
☒	☐ ChIP-seq
☐	☒ Flow cytometry
☒	☐ MRI-based neuroimaging

Antibodies

Antibodies used

Intracellular cytokine staining: CD28/CD49 (Cat# 9035982, Lot# 2235086; BD Biosciences), anti-CD3-BUV395 (Cat# 564001, Lot# 1307923; BD Biosciences), anti-CD4-AF700 (Clone RPA-T4, Cat# 300526, Lot# B368193; BioLegend), anti-CD19-BV510 (Clone HIB19, Cat# 302242, Lot# B334934; BioLegend), anti-CD14-BV510 (Clone M5E2, Cat# 301842, Lot# B349984; BioLegend), anti-CD8-APC-Cy7 (Clone SK1, Cat# 344714, Lot# B281019; BioLegend), anti-CCR7-AF647 (Clone G043H7, Cat# 353218, Lot# B357238; BioLegend), anti-CD45RO-FITC (Clone UCHL1, Cat# 304242, Lot# B362185; BioLegend), anti-IFN γ-PE-Cy7 (Clone B27, Cat# 506518, Lot# B359850; BioLegend), anti-TNF α-PE/Dazzle™ 594 (Clone MAb11, Cat# 502945, Lot# B266375; BioLegend) and IL 2-PerCP-Cy5.5 (Clone MQ1-17H12, Cat# 500322, Lot# B312837; BioLegend)
IFN-γ ELISpot: anti-IFN-γ (Clone 1-D1K, Cat# 3420-6, Batch# 56.3; Mabtech), anti-IFN-γ-HRP (Clone 7-B6-1, Cat# 3310-10, Batch# 44127.2-10; Mabtech)
IGRA assay: anti-IFN-γ-Biotin (Kit# EQ 6841-9601, Lot# E200925BW, EUROIMMUN)
IgG Elispot: anti-IgG coating antibody (Clone MT91/145, Batch 11.3, Kit# 3850-2A, Mabtech), anti-IgG-Biotin (Clone MT78/145, Batch 7.5, Kit# 3850-2A, Mabtech)
Peptide microarrays: anti-human IgA (alpha chain) -Dylight 800 (Cat# 609145006, Lot# 32937; Rockland Inc.), anti-human IgG (Fc fragment) -Dylight 680 (Cat# A80304D6, Lot# A80304D66; Bethyl)
Bead-based multiplex immunoassay: anti-IgG-PE (polyclonal, Cat# HC19-PEIGG, Lot# C19PEG-1K), anti-IgA-PE (polyclonal, Cat# HC19-PEIGA, Lot# PEIGA-1), anti-IgM-PE (polyclonal, Cat# HC19-PEIGM, Lot# HC19-1K), anti-IgG1-PE (Clone 4E3, Cat# SBA-9052-09, Lot# H0321-V611B), anti-IgG2-PE (Clone HP6002, Cat# SBA-9070-09, Lot# I1121-WA81), anti-IgG3-PE (Clone HP6050, Cat# SBA-9210-09, Lot# F5821-SM71C), anti-IgG4-PE (Clone HP6025, Cat# SBA-9200-09, Lot# F0421-XB21B)

Validation

All antibodies were acquired commercially and were validated by the manufacturer.

Eukaryotic cell lines

Policy information about cell lines and Sex and Gender in Research

Cell line source(s)

Vero C1008 cells (ATCC, Cat. no. CRL-1586, RRID:CVCL_0574)

Authentication

Cell line was authenticated by DNA profiling of eight polymorphic regions of short tandem repeats in 2016 by the Leibniz-Institut DSMZ GmbH.

Mycoplasma contamination

When the cells were thawed and discarded, the cell line tested negative for mycoplasma contamination.

Commonly misidentified lines
(See ICLAC register)

NA

Clinical data

Policy information about clinical studies
All manuscripts should comply with the ICMJE guidelines for publication of clinical research and a completed CONSORT checklist must be included with all submissions.

Clinical trial registration

ClinicalTrials.gov. (NCT04569383; NCT04895449)

Study protocol

The clinical trial protocols are available upon request from the corresponding author.

Data collection

The MVA phase 1 clinical trials were single-center studies conducted in Hamburg, Germany. For the MVA-S/mRNA study cohort, the first study participant was included in October 2020, and the last sampling time point included into this manuscript was in August 2021. For the MVA-ST and mRNA/MVA-ST cohorts, the first participants were included in July and December 2021, respectively, and the last sampling time points were in April and June 2022. The clinical research organization Clinical Trial Center North in Hamburg performed the operative and regulatory project management of the trial; the University Medical Center Hamburg-Eppendorf was the Sponsor or this investigator-initiated trial. The control cohorts were recruited and sampled between December 2020 and April 2022 in Hamburg, Germany. All data collection was performed in Hamburg, Germany.

Outcomes

No primary and secondary endpoints are reported in this manuscript.

Plants

Seed stocks

Report on the source of all seed stocks or other plant material used. If applicable, state the seed stock centre and catalogue number. If plant specimens were collected from the field, describe the collection location, date and sampling procedures.

Novel plant genotypes

Describe the methods by which all novel plant genotypes were produced. This includes those generated by transgenic approaches, gene editing, chemical/radiation-based mutagenesis and hybridization. For transgenic lines, describe the transformation method, the number of independent lines analyzed and the generation upon which experiments were performed. For gene-edited lines, describe the editor used, the endogenous sequence targeted for editing, the targeting guide RNA sequence (if applicable) and how the editor was applied.

Authentication

Describe any authentication procedures for each seed stock used or novel genotype generated. Describe any experiments used to assess the effect of a mutation and, where applicable, how potential secondary effects (e.g. second site T-DNA insertions, mosiacism, off-target gene editing) were examined.

Flow Cytometry

Plots

Confirm that:

☒ The axis labels state the marker and fluorochrome used (e.g. CD4-FITC).

☒ The axis scales are clearly visible. Include numbers along axes only for bottom left plot of group (a 'group' is an analysis of identical markers).

☒ All plots are contour plots with outliers or pseudocolor plots.

☒ A numerical value for number of cells or percentage (with statistics) is provided.

Methodology

Sample preparation

Whole blood was collected in EDTA vacutainers. After centrifugation, plasma was removed and stored at -80°C. PBMCs were isolated by density-gradient centrifugation using Ficoll-Histopaque (Sigma) or SepMate™ (Stemcell), cryopreserved, and stored in liquid nitrogen. For the flow cytometry assay, PBMCs were thawed, rested overnight, and were then stimulated with S peptides (2.5 ug/ml) for 7h at 37°C in the presence of Golgi-Plug, Golgi-Stop, and anti CD28/CD49 (#9035982; BD Biosciences) in 96 well V bottom plates (Sarstedt). For each sample, cells incubated with an equimolar amount of DMSO (0.1 %) and Phorbol-12-myristate-13-acetate (50 ng/ml), and ionomycin (0.5 ug/ml) served as negative and positive controls, respectively. Cells were then washed and stained with an antibody mix of anti-CD3-BUV395 (#564001; BD Biosciences), anti-CD4-AF700 (#300526; BioLegend), anti-CD19-BV510 (#302242; BioLegend), anti-CD14-BV510 (#301842; BioLegend), anti-CD8-APC-Cy7 (#344714; BioLegend), anti-CCR7-AF647 (#353218; BioLegend), anti-CD45RO-FITC (#304242; BioLegend), and Zombie Aqua™ Fixable Viability Kit (#423101; BioLegend) in FACS buffer [PBS supplemented with 2 % FBS and 2 mM EDTA] for 15 minutes at 37°C. Subsequently, cells were fixed (eBioscience™), washed, and stained with intracellular markers anti-IFN γ-PE-Cy7 (#506518; BioLegend), anti-TNF α-PE/Dazzle™ 594 (#50296; BioLegend) and IL 2-PerCP-Cy5.5 (#500322, BioLegend) in PERM buffer (eBioscience™) at RT for 15 minutes. Samples were stored in FACS buffer at 4°C and analyzed on the BD Fortessa the following day.

Instrument

BD LSRFortessa™ Cell Analyzer

Software

BD FACSDiva software; FlowJo (v.10.8.1)

Cell population abundance

1,000,000 PBMCs of each sample were used for the intracellular cytokine staining assay. At least 50,000 live CD3+ events were recorded for each sample. Sample quality was confirmed by using PMA/Iono and CEF stimulaton as positive controls, and unstimulated cells as negative controls for each sample.

Gating strategy

Cytokine-secreting T cells were identified by excluding acquisition errors with the time gate, then gating lymphocytes based on the FSC-A and SSC-A channels. Douplets were excluded by FSC-A vs FSC-H and then SSC-A vs SSC-H gating. Dead cells, granulocytes and B cells were further excluded using a dump channel (consisting of viability dye, and antibodies against CD19 and CD14). T cells were identified as being CD3+ and CD4+ or CD8+. Memory T cells were identified by excluding naive CCR7+CD45RO- cells. Cytokine positive responses are shown after subtraction of background responses detected in the corresponding unstimulated sample. All gates were set based on FMO controls and positive control samples (PMA/iono or CEF stimulation). Boolean gates were used to analyze polyfunctionality (expression of 1, 2, or 3 cytokines).

☒ Tick this box to confirm that a figure exemplifying the gating strategy is provided in the Supplementary Information.
